# Walnut Intake Interventions Targeting Biomarkers of Metabolic Syndrome and Inflammation in Middle-Aged and Older Adults: A Systematic Review and Meta-Analysis of Randomized Controlled Trials

**DOI:** 10.3390/antiox11071412

**Published:** 2022-07-21

**Authors:** Letiția Mateș, Daniela-Saveta Popa, Marius Emil Rusu, Ionel Fizeșan, Daniel Leucuța

**Affiliations:** 1 Department of Toxicology, Faculty of Pharmacy, Iuliu Hatieganu University of Medicine and Pharmacy, 8 Victor Babes, 400012 Cluj-Napoca, Romania; micu.letitia@umfcluj.ro (L.M.); ionel.fizesan@umfcluj.ro (I.F.); 2 Department of Pharmaceutical Technology and Biopharmaceutics, Faculty of Pharmacy, Iuliu Hatieganu University of Medicine and Pharmacy, 8 Victor Babes, 400012 Cluj-Napoca, Romania; 3 Department of Medical Informatics and Biostatistics, Faculty of Medicine, Iuliu Hatieganu University of Medicine and Pharmacy, 8 Victor Babes, 400012 Cluj-Napoca, Romania; dleucuta@umfcluj.ro (D.L.)

**Keywords:** nuts, tree nuts, nut consumption, aging, age-related diseases, cardiometabolic markers, antioxidants, inflammation, lipid profile, diabetes

## Abstract

Biomarkers of metabolic syndrome and inflammation are pathophysiological predictors and factors of senescence and age-related diseases. Recent evidence showed that particular diet components, such as walnuts rich in antioxidant bioactive compounds and with a balanced lipid profile, could have positive outcomes on human health. A systematic search in PubMed, EMBASE, Cochrane Library, Scopus, and ClinicalTrials.gov databases was performed to retrieve randomized controlled trials published from the beginning of each database through November 2021, reporting on the outcomes of walnut consumption over 22 metabolic syndrome and inflammatory markers in middle-aged and older adults. The search strategy rendered 17 studies in the final selection, including 11 crossover and 6 parallel trials. The study revealed that walnut-enriched diets had statistically significant decreasing effects for triglyceride, total cholesterol, and LDL cholesterol concentrations on some inflammatory markers and presented no consequences on anthropometric and glycemic parameters. Although further studies and better-designed ones are needed to strengthen these findings, the results emphasize the benefits of including walnuts in the dietary plans of this age group.

## 1. Introduction

Metabolic syndrome (MetS) conditions, chronic, low-grade inflammation, and oxidative stress are significant risk factors for morbidity and mortality with higher prevalence in the aging population [1]. These pathophysiological components increase the probability of age-associated diseases, including cardiovascular disease (CVD), type 2 diabetes (T2D), cognitive impairment, neurodegenerative disorders, or cancer [2,3]. Compelling evidence demonstrates that inflammatory markers, such as serum C-reactive protein (CRP), tumor necrosis factor-alpha (TNF-α), interleukin-1β (IL-1β), interleukin-6 (IL-6), intercellular adhesion molecule-1 (ICAM-1), and vascular cell adhesion molecule-1 (VCAM-1), are predictors and factors in cellular senescence and chronic inflammatory conditions [4].

Human and animal examinations suggested that plant matrices rich in antioxidant and anti-inflammatory compounds could prove efficient in protecting against oxidative stress and excessive inflammation [5,6,7,8]. Extensive research examined the effects of plant-based diets on various health outcomes [9,10]. Tree nuts, important plant nutrient sources, are rich in monounsaturated fatty acids (MUFAs) and polyunsaturated fatty acids (PUFAs), tocols, phytosterols, and polyphenols, essential bioactive phytochemicals with demonstrated antioxidant properties [11]. Several studies consistently showed the antioxidant activity and anti-inflammation potential of the active compounds from tree nut kernels or by-products and their association with a reduced risk for CVD, T2D, cancer, and all-cause mortality [12,13,14,15]. Of the different types of nuts, walnuts are especially rich in linoleic acid (18:2n–6), α-linolenic acid (ALA) (18:3n–3), polyphenols, L-arginine, and magnesium [16], a unique phytochemical profile responsible for many beneficial effects. It was suggested that walnuts might modulate neuroplasticity, neuroprotection, and vasodilation of brain arteries [17] or decrease cancer growth, reduce metastasis, and increase cancer cell death via altering tumor gene expression [18].

Several studies have previously linked walnut intake with lipid profile beneficial effects and lowering of reactive oxygen species (ROS) and inflammatory markers in different age groups [19,20,21].

Contrary to the above results, a recent meta-analysis found no associations between walnut consumption and glucose homeostasis as well as inflammation [22]. Moreover, increasing dietary ALA intake did not affect inflammatory markers [23].

Based on these conflicting conclusions, we aimed to perform a systematic review and meta-analysis of randomized controlled trials (RCTs) to thoroughly assess the data concerning the effects of walnut intake on selected markers of inflammation and metabolic syndrome in mature adults. As the exact etiology of chronic inflammation and its potential causal function in unfavorable health outcomes are mostly unknown, research on markers of inflammation and the identification of pathways to control age-associated inflammation is of great relevance for the prevention of inflammation and management of age-associated diseases. To the best of our knowledge, this is the first meta-analysis conducted on the impact of walnut consumption on markers of inflammation and metabolic syndrome in middle-aged and older adults.

## 2. Materials and Methods

The current meta-analysis was performed following the PRISMA criteria guidelines [24]. The registration code is INPLASY202260058, with DOI 10.37766/inplasy2022.6.0058, https://inplasy.com/inplasy-2022-6-0058/ (accessed on 13 June 2022).

### 2.1. Eligibility Criteria

Our systematic review included (1) randomized controlled parallel or crossover trial studies that compared the effect of (2) walnuts consumption, (3) with a minimum 3-week intervention period in (4) middle-aged and older adults (≥40 years of age or mean age ≥ 50 years), (5.a) on MetS biomarkers, including waist circumference (WC), body weight (BW), body mass index (BMI), systolic blood pressure (SBP), diastolic blood pressure (DBP), triglyceride (TG), total cholesterol (TC), high-density lipoprotein (HDL) cholesterol (HDL-C), low-density lipoprotein (LDL) cholesterol (LDL-C), fasting blood glucose (FBG), and glycosylated hemoglobin A1c (HbA1c), as well as on the insulin resistance index (homeostatic model assessment for insulin resistance (HOMA-IR) and insulin), and on (5.b) inflammatory biomarkers, including C-reactive protein (CRP), high-sensitivity C-reactive protein (hs-CRP), interferon gamma (IFN-γ), E-selectin, VCAM-1, ICAM-1, TNF-α, and interleukins (IL-6 and IL-1β), as primary or secondary outcomes. We excluded: (1) abstracts, narrative reviews, comments, opinions, methodological papers, editorials, letters, observational studies, conference abstracts, case studies, in vitro studies, non-human, with a mechanistic, non-stochastic modeling, or any other publications lacking primary data and/or explicit method explanations; (2) irrelevant interventions (walnuts oil, walnut extract, nut mix); (3) irrelevant comparisons (compulsory comparison); (4) publications with full text not available; (5) duplicate studies or databases; and (6) publications in languages that were not known.

### 2.2. Information Sources

We performed a systematic literature search in PubMed, EMBASE, Cochrane Library, Scopus, and ClinicalTrials.gov databases for controlled trials describing the effects of walnut consumption on metabolic syndrome and inflammatory biomarkers in mature adults from the inception of each database through November 2021. The literature search had no language constraint. To ensure thorough research, the bibliographies of the included studies and current reviews were also screened.

### 2.3. Search Strategy

To search the databases, we used a combination of free-text words, along with their synonyms, singular and plural forms, thesaurus words (Medical Subject Headings for PubMed, and Emtree for EMBASE), and abbreviations concerning the following concepts: (1) walnuts; (2) inflammatory biomarkers, C-reactive protein, interleukins, tumor necrosis factor, vascular cell adhesion molecule, intercellular adhesion molecule, selectin, adiponectin, adhesion molecules; (3) metabolic syndrome, waist circumference, weight, body mass index, systolic and diastolic blood pressure, triglycerides, total, HDL-C and LDL-C, glycemia, HbA1c, insulin resistance, HOMA-IR, insulin; and (4) randomized controlled trial. The entire search strategy for each database is presented in Supplementary Table S1.

### 2.4. Selection Process

Three investigators (D.L., L.M., and D.-S.P.) independently checked the titles and abstracts for relevant articles. Following that, the full texts of those that looked to satisfy the selection criteria were retrieved for further selection. The same investigators independently checked each full text. In the event of a disagreement, the studies were debated until a consensus was reached. In the instance of multiple publications from the same trial, only the most recent or informative article was selected.

### 2.5. Data Items

Data regarding the outcomes were extracted in a spreadsheet Microsoft (Microsoft Office 365, MS, Redmond, WA, USA) Excel file: (1) inflammatory biomarkers, C-reactive protein, interleukins, tumor necrosis factor, vascular cell adhesion molecule, intercellular adhesion molecule, selectin, adiponectin, adhesion molecules; (2) metabolic syndrome, waist circumference, weight, body mass index, systolic and diastolic blood pressure, triglycerides, total, HDL and LDL cholesterol, glycemia, HbA1c, insulin resistance, HOMA-IR, insulin. For each variable, the baseline, final, and differences between baseline and final observations were extracted, as well as the differences between the interventions regarding the final values or the differences between baseline and final observations.

Furthermore, data regarding study characteristics were extracted in a spreadsheet file: country, study design, exposure period, washout period, participants number in each group, health status, age, female percentage, walnut intervention quantity and type, control intervention, and the outcome of interest.

Other investigators than those who extracted the initial full-text articles rechecked the extracted data.

### 2.6. Study Risk of Bias Assessment

The risk of bias was assessed for each selected article using the Risk of Bias 2 Tool from Cochrane [25] in duplicate, and the disagreements were resolved by discussion.

### 2.7. Effect Measures

For all the outcomes, we used the standardized mean difference in the synthesis and presentation of results.

### 2.8. Synthesis Methods

We calculated the means and standard deviations for each variable utilized in the meta-analysis. When the standard deviation (SD) was not known, it was calculated using the standard error (SE) or mean, medians and interquartile ranges (IQRs), confidence intervals (CIs), or *p*-values, according to Cochrane Handbook recommendations [26]. The differences between the intervention groups in terms of changes (baseline–final values) were the preferred values in analyses. Otherwise, we computed the differences between the final values if these data were unavailable for the changes. We calculated the mean difference (between changes or between final values) and the SE for each trial, either parallel or crossover, in order to be able to pool the results from both designs, as recommended by Elbourne et al. [27]. The meta software was used to perform meta-analyses on these mean differences and SE [28]. The standardized mean difference along with 95% CI was computed for each variable, using the random effects model due to clinical heterogeneity between the trials. The Paule–Mandel estimator was used to estimate the between-study variance within the inverse variance method. The statistical heterogeneity between the studies was assessed with χ2-based Q-test and I^2^. Next, high leverage studies were identified with the dmeta package [29]. Furthermore, subgroup analyses were performed for risk of bias, trial design, exposure duration, walnut quantity, health status, control group, and age, in case more than ten studies were available. To assess the robustness of the results, a leave-one-out sensitivity analysis was used. If the *p*-value was less than 0.05, statistical significance was assumed. For all analyses, the R environment for statistical computing and graphics (R Foundation for Statistical Computing, Vienna, Austria) version 4.1.2 [30] was used.

### 2.9. Quality Assessment

We used the Cochrane Collaboration’s Risk of Bias Tool 2 to examine the selected studies: the parallel trial version for the parallel studies and the crossover trial version for the crossover studies.

### 2.10. Reporting Bias Assessment

In case there were more than ten studies available to analyze a variable of interest, a funnel plot and the Egger test were performed to assess the presence of publication bias.

## 3. Results

**Figure 1:**
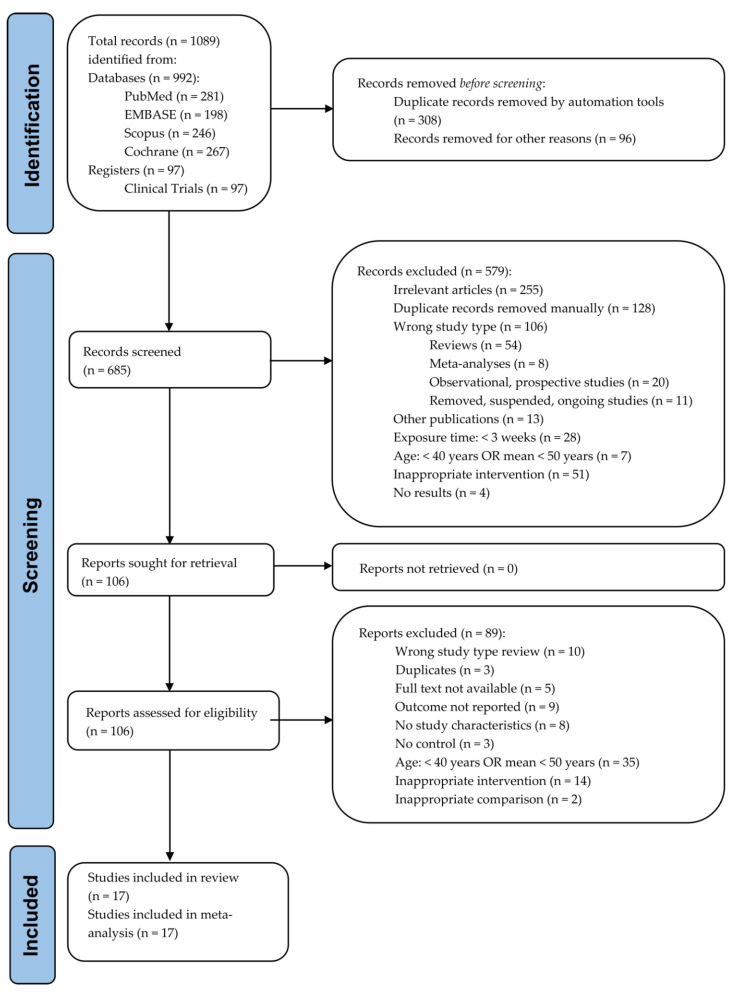
PRISMA flow diagram of study selection.

A total of 685 articles were considered from the systematic search and review of relevant reference lists. After applying exclusion criteria, 17 articles were included in the systematic review and meta-analysis. The procedure of study inclusion and exclusion is shown in Figure 1. The characteristics of the included studies are revealed in Table 1 and Supplementary Table S2.

### 3.1. Metabolic Syndrome Biomarkers

The effects of walnut-enriched diets on the biomarkers of MetS and inflammation are presented in Table 2.

#### 3.1.1. Triglycerides

**Figure 2:**
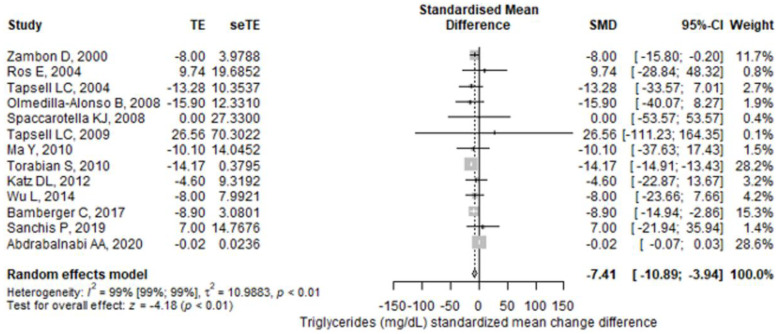
Forest plot for triglycerides (mg/dL) standardized mean change difference. TE—treatment effect; seTE—the standard error of the treatment effect; SMD—standardized mean difference; CI—confidence interval.

**Figure 3:**
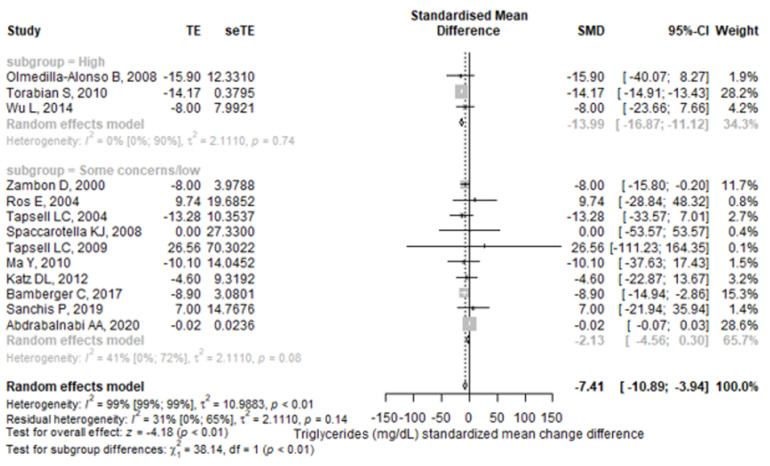
Forest plot for triglycerides (mg/dL) standardized mean change difference compared with subgroup analyses for risk of bias. TE—treatment effect; seTE—the standard error of the treatment effect; SMD—standardized mean difference; CI—confidence interval.

From the selected studies, thirteen studies reported TG values. The meta-analysis found a higher reduction in TG values in the walnut group compared to the control group (SMD = −7.41 (95% CI: −10.89–−3.94), *p* < 0.001) (Figure 2). There was a significant heterogeneity between the studies, I^2^ of 99.1% (95% CI: 99–99.3%), and the Q test for heterogeneity gave *p* < 0.001. The results remained statistically significant after performing a leave-one-out sensitivity analysis for each study. The studies that influenced the final result the most were Tapsell et al. (2009) [36] and Abdrabalnabi et al. [45]; their removal brought the I^2^ to values lower than or equal to 7%. A subgroup analysis found that studies with a high risk of bias had higher reductions in TG values than studies with some concerns or low risk of bias, the pooled result remaining statistically significant only for high risk of bias studies (Figure 3). The subgroup analyses regarding treatment exposure duration, study population health, and diet showed statistically significant results for each subgroup (Supplementary Figures S1–S4). The subgroup exposed to walnut portions greater than 42 g/day had statistically significant results, but for those having lower portions, the pooled result lost its significance (Supplementary Figure S5). Finally, for the crossover studies subgroup, the final result was statistically significant, but not for the parallel studies subgroup (Supplementary Figure S6).

#### 3.1.2. Total Cholesterol, LDL, and HDL Cholesterol

**Figure 4:**
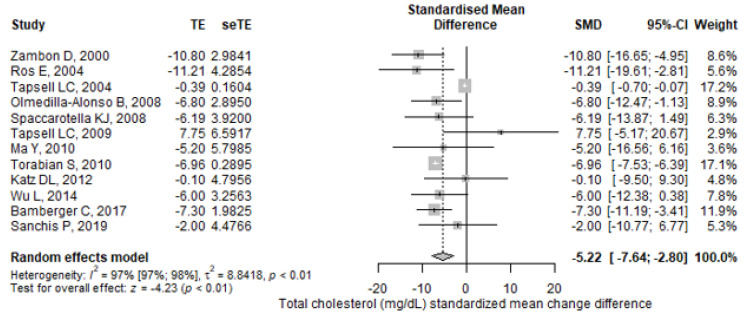
Forest plot for total cholesterol (mg/dL) standardized mean change difference. TE—treatment effect; seTE—the standard error of the treatment effect; SMD—standardized mean difference; CI—confidence interval.

Results for mean differences in TC and LDL-C between intervention and control groups were reported in 12 trials (10 crossover and 2 parallel). We noticed significantly lower values for TC concentrations in walnut-enriched diets compared to control diets (SMD = −5.22, 95% CI: −7.64–−2.8), *p* < 0.001) (Figure 4), with significant heterogeneity between the experiments (I^2^ = 99.1%; 95% CI: 99–99.3%, *p*-heterogeneity < 0.001).

**Figure 5:**
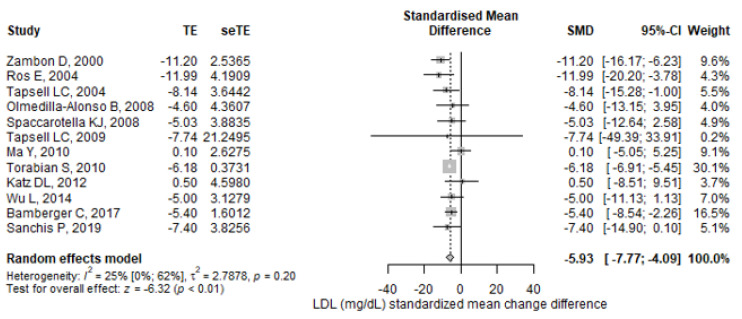
Forest plot for LDL cholesterol (mg/dL) standardized mean change difference. TE—treatment effect; seTE—the standard error of the treatment effect; SMD—standardized mean difference; CI—confidence interval.

Similarly, the meta-analyzed SMD displayed a significantly greater reduction in LDL-C concentrations with the walnut diets than with the control diets (SMD = −5.93; 95% CI: −7.77–−4.09, *p* < 0.001) (Figure 5), but without significant heterogeneity (I^2^ = 24.8%; 95% CI: 0–61.8%, *p*-heterogeneity = 0.2).

Sensitivity analyses showed that the outcomes remained statistically significant after removing one study at a time for both parameters. The reports with the highest influence on the final effects were Tapsell et al. (2004) [33] and Tapsell et al. (2009) [36] for TC, while for LDL-C they were Torabian et al. [38] and Tapsell et al. (2009) [36].

In the subgroup analyses, for TC and LDL-C parameters, walnut diets had statistically significant effects in high risk and some concern studies for risk of bias, as well as for exposure duration, walnut quantity, population health, and diet in both study designs, parallel and crossover. The results remained significant for the participant subgroup with ages over 40 years (Supplementary Figures S7–S19).

Fourteen controlled trials documented results for HDL-C. There were no statistically significant changes in HDL-C concentrations between the walnut and the control diets (SMD = −0.18; 95% CI: −0.59–0.22, *p* = 0.375). However, a significant heterogeneity was reported (I^2^ = 47.4%; 95% CI: 0–72.4%, *p*-heterogeneity = 0.029) (Supplementary Figure S20).

#### 3.1.3. Anthropometric Markers

WC, BMI, and BW changes were reported in three, five, and eight trials, respectively. On these parameters, individual trials did not show significant differences compared to the control after following a walnut-enriched diet (WC SMD = −0.14; 95% CI: −0.8–0.51, *p* = 0.671; BMI SMD = 0.11; 95% CI: −0.11–0.34, *p* = 0.326; BW SMD = 0; 95% CI: −0.4–0.39, *p* = 0.987). A significant heterogeneity was observed only for BMI (*p* = 0.028) (Supplementary Figures S21–S23).

#### 3.1.4. Blood Pressure

The effect on blood pressure was analyzed in eight studies (six crossover and two parallel). Walnut-enhanced diets did not significantly modify SBP (SMD = −0.85; 95% CI: −4.48–2.77, *p* = 0.644) or DBP (SMD = −0.34; 95% CI: −1.68–1, *p* = 0.62), with significant heterogeneity for SBP (I^2^ = 64.4%; 95% CI: 24–83.4%, *p*-heterogeneity = 0.006) (Supplementary Figures S24 and S25).

#### 3.1.5. Glycemic Biomarkers

Similarly, no significant reductions were detected for FBG, HbA1c, HOMA-IR, and insulin assessed in six, four, three, and four studies, respectively. Compared with control diets, walnut-enriched diets accounted for a non-significant decrease of these glycemic markers (FBG SMD = 0.01; 95% CI: 0–0.02, *p* = 0.088; HbA1c SMD = 0.08; 95% CI: −0.04–0.2, *p* = 0.196; HOMA-IR SMD = 0.03; 95% CI: −0.44–0.5, *p* = 0.891; insulin SMD = 0.91; 95% CI: −2.16–3.98, *p* = 0.561). The Q test for heterogeneity gave a significant value only for insulin (*p* = 0.034) (Supplementary Figures S26–S29).

### 3.2. Inflammatory Biomarkers

In the meta-analysis of the inflammatory markers, the walnut consumption revealed no significant influence on CRP (SMD = −0.37; 95% CI: −1.39–0.65, *p* = 0.478) and hs-CRP (SMD = −0.01; 95% CI: −0.12–0.11, *p* = 0.903) (Supplementary Figures S30 and S31).

For the other studied inflammatory biomarkers, the walnut diet showed significant changes (IFN-γ SMD = −1.26; 95% CI: −2.01–−0.51, *p* < 0.001; IL-6 SMD = −0.18; 95% CI: −0.00–−0.03, *p* < 0.001; IL-1β SMD = −0.1; 95% CI: −0.16–−0.04, *p* < 0.001; TNF-α SMD = −0.31; 95% CI: −0.54–−0.08, *p* = 0.009; E-selectin SMD = −2.57; 95% CI: −4.09–−1.05, *p* < 0.001), but the publication bias test and the heterogeneity could not be calculated since there was only one assessed study (Supplementary Figures S32–S36).

The endothelial adhesion molecules, ICAM-1 and VCAM-1, could not be computed since the algorithm did not converge when the study of Canales et al. [39] was included in the analysis. When we excluded this analysis, the results based on the studies of Wu et al. and Cofan et al. [41,47] were not statistically significant.

### 3.3. Quality Assessment

The results obtained after quality (risk of bias) assessment for the six parallel and eleven crossover studies are presented in the Supplementary Materials (Supplementary Figures S37 and S38). Several papers [43,44,46,47] analyzed data from the same study, Walnuts and Healthy Aging (WAHA), and for the quality assessment they were considered as only one.

Concerning the randomization process domain, eleven studies (79%) had some concerns of bias, and three were at low risk of bias. The randomization generation method was presented in five studies. Only one study mentioned allocation concealment. Only two studies (14%) explained how randomization was undertaken. For crossover studies, seven had no information to assess the start of clinical study baseline differences, and three probably did not have differences, while for parallel trials, all four probably did not have differences.

For crossover trials, we assessed the risk of bias arising from period and carryover effects. Four studies had some concerns of bias, and seven were at low risk of bias. Five studies had a similar number of subjects allocated to the interventions. Two studies probably did not have important differences in the number of subjects allocated to the interventions. The other studies reported no information. Five studies did not analyze whether the period effect was verified. All studies had sufficient time for the disappearance of any carryover effects before the outcome assessment in the second period.

Regarding deviations from the intended interventions, four studies were at high risk of bias, six had some concerns of bias, and four were at low risk of bias. Although not mentioned in all trials, participants, caregivers, and those administering interventions were likely all aware of the assigned intervention, except for one study where the investigator was blinded to the intervention. The studies did not mention whether deviations from the intended intervention arose due to trial context, except in one study where those deviations probably did not affect the outcome. Five studies mentioned or it could be deducted that they used an intention-to-treat analysis. Five studies gave no information about the use of an intention-to-treat analysis, and the other four stated or it could be deducted that they used a per-protocol analysis. Only one study was impacted by the lack of intention-to-treat analysis of the results.

Moreover, only one study had some concern of bias with respect to missing outcome data domain; the others had a low risk of bias. Seven studies probably had data for all or nearly all randomized participants. Six studies had important percentages of subjects that dropped out. One study did not report anything about missing data. No study provided missing data analysis or sensitivity analyses to demonstrate that missing data did not skew the results. In all the research, it is more likely to conclude that the missingness of the outcomes was unrelated to its genuine value.

Concerning the measuring of the outcome domain, all the studies were at low risk of bias. All of the studies used the same instruments and standard and exact measuring methods to test the outcomes at the same time points throughout their research (laboratory assays or anthropometric measurements). In the case of laboratory measurements, it is likely the measurement was blinded (only three studies reported it). It is unlikely that knowing the intervention would influence the measurement.

Considering the selection of the reported result domain, all the studies had a low risk of bias. Two studies had variables of interest for our review specified as the primary endpoint in the research protocol. Four studies had research protocols published before their study but with different primary endpoints compared to our review. Only one instrument and one statistical analysis approach were employed in all of the investigations for each variable of interest.

Overall, four studies were considered at high risk of bias, and the others showed some concerns of bias.

### 3.4. Reporting Bias Assessment

The Egger test yielded non-statistically significant findings for all of the outcomes of interest when it was used to examine the presence of publication bias. Moreover, funnel plots were not indicative of asymmetry either.

## 4. Discussion

To the best of our knowledge, the current study is the first systematic review and meta-analysis to focus on comprehensively analyzing the evidence to date regarding the effects of walnut-enriched diets on biomarkers of MetS and inflammation in middle-aged and older adults.

Walnut is considered a nutraceutical dietary source due to the high content of good fatty acids, such as MUFA and omega-3 PUFA, its nutritional value, the high antioxidant phytochemical content, and its beneficial effects on human health.

In the present meta-analysis, we assessed the results of seventeen randomized clinical trials that analyzed the impact of walnut-enriched diets. Our findings showed that walnut-enriched diets significantly decreased TG, TC, and LDL-C concentrations, while HDL-C level was not significantly affected. No significant changes were noticed on anthropometric, cardiometabolic, and glycemic indices after higher walnut consumption. Moreover, the inflammatory biomarkers did not record statistically significant results.

Considering the evidence from recent meta-analyses, nut consumption [48,49,50] and walnut-enriched diets [22,51] are negatively associated with specific biomarkers of MetS and inflammation in different age groups.

Regarding the duration of exposure to treatment (in a range between 4 weeks and 2 years), the health of the studied population (healthy people, hypertensive, hypercholesterolemic, or T2D patients), and the diet (mostly Western-type or habitual diet, without walnuts), each subgroup presented statistically significant results. Different doses of walnut showed that the subgroup exposed to walnut portions greater than 42 g per day had statistically significant results. This result reinforces the Food and Drug Administration (FDA) [52] recommendation for the inclusion of 42 g (1.5 ounces) of walnuts in the daily diet and differs from the conclusions of another meta-analysis, which states that the TG lowering effects reach a plateau at doses higher than 20 g [53].

Our meta-analysis identified a statistically significant reduction of TG values (*p* < 0.001) in walnut consumption groups compared to control groups in the thirteen trials analyzed for this marker. Furthermore, it showed statistically significant decreases in terms of TC and LDL-C levels. Analyzing the twelve studies reporting results for mean differences in TC and LDL-C, we noticed significantly lower values for TC concentrations in walnut-enriched diets (*p* < 0.001) compared to control diets. Similarly, we registered a significantly greater reduction in LDL-C concentrations with the walnut diets (*p* < 0.001) than with the control diets. The statistically significant beneficial effects in the lipid profile noticed after walnut-enriched diets have the potential of decreasing the age-related disease risks for the age category targeted in this meta-analysis.

Several observational studies obtained the same answers. A cross-sectional study analyzing data from three large US prospective cohort studies concluded that an increase of 0.5 servings (~14 g) per day in walnut consumption was significantly associated with 17% lower CVD risk and 20% lower stroke risk [54]. After assessing the same data but with slightly different covariates, another study found that consuming at least one serving (~28 g) of walnuts per week was linked with 19% lower CVD risk and 17% lower stroke risk, in addition to a 21% decrease in the risk of CHD [55]. Similarly, a recent systematic review and meta-analysis of prospective studies revealed that higher walnut intake was associated with lower risks of CVD and CHD incidence [56]. Moreover, data from two large prospective cohort studies associated higher walnut consumption with a lower CVD risk and mortality and a greater life expectancy among U.S. older adults [57]. The PREDIMED study also disclosed a significantly lower risk of stroke in participants who consumed 30 g of mixed nuts (including 15 g of walnuts) per day compared with a no-nut consumer group [58].

Based on our results, the improvement of the lipid profile and decrease of oxidative stress and inflammation are primary mechanisms of walnut intake against CVD. Furthermore, bioactive compounds found in walnuts, both hydrophilic and lipophilic, could protect against MetS complications and CVD [15].

Thereby, polyphenols, hydrosoluble micronutrients found in walnuts such as quercetin and its glycosides, ellagic acid and ellagitannins, and cyanidin and proanthocyanidins [59] exert their antioxidant action through multiple mechanisms, including the activation of the Nrf2/ARE (nuclear factor erythroid 2-related factor 2/antioxidant response element) pathway. By this pathway, polyphenols increase the activity of some antioxidant and detoxifying enzymatic systems and down-regulate the nuclear factor kappa B (NF-кB) pathway that is directly implicated in the inflammatory response. Tocopherols and tocotrienols, as well as n-3 PUFAs and n-6 PUFAs and other lipophilic antioxidants from walnuts, can also inhibit the NF-кB pathway by activation of Nrf2/ARE. By preventing oxidation of LDL, antioxidants improve the lipid profile, preventing and reducing the formation of atherosclerotic plaques and the risks for CVD [11]. Melatonin, found in minute quantities in walnuts (3.5 ± 1.0 ng/g), holds antioxidant and anti-inflammatory properties, with CV protection [16]. Moreover, phytosterols from walnuts can lower LDL-C levels. They are more hydrophobic than cholesterol and can dislocate cholesterol from intestinal micelles and reduce LDL-C absorption. In combination with n-3 PUFAs, phytosterols show both complementary and synergistic lipid-lowering effects [16].

In our study, the effect on blood pressure was analyzed in eight trials. Neither SBP nor DBP was significantly modified by walnut-enhanced diets, confirming the results of previous analyses [53,55,60]. Furthermore, our study did not show statistically significant changes in terms of glycemic markers, which also corroborated prior studies [22,51,53]. After following walnut-enriched diets, the anthropometric parameters did not show significant differences compared to the control. These results were consistent with those obtained in former works [49,61].

Low-grade chronic inflammation, referred to as inflammaging in the older population, plays a key role in atherosclerosis, while inflammation biomarker concentrations can predict future T2D or CVD events [62]. The results of our study showed no significant effects of walnut intake on inflammatory markers. These findings concur with recently published data showing that the hs-CRP level was not influenced by walnut consumption [53]. Moreover, our findings agree with a recently published meta-analysis of both interventional and observational studies, which established that walnut intake had no statistical significance on glucose homeostasis and inflammation [22]. In contrast, observational studies found that nut consumption was inversely associated with inflammatory markers [63]. These findings might point to other types of nuts being responsible for these positive effects. However, Cofán et al. (2020) [47] are the only researchers who have studied several biomarkers of inflammation in correlation with a walnut-diet and found statistically significant reduction for IL-6, IFN-γ, IL-1β, TNF-α, and E-selectin, but not for hs-CRP and adhesion molecules VCAM-1 and ICAM-1. These results are noteworthy, but further clinical trials are needed to confirm them.

The negative relationship between walnut intake and MetS pathophysiology may also be attributed to the antioxidant and anti-inflammatory activity of vitamin E [62] and other antioxidant phytochemicals found in walnuts [64].

Similar to other studies, our meta-analysis presents several limitations. The most important limitation concerns the risk of bias present in the selected studies. Blinding participants and personnel in the case of walnut eating is clearly challenging, particularly for participants, and was not performed in the studies. Nonetheless, because the majority of the outcomes of interest are objective laboratory measurements, this methodological shortcoming is less likely to impact the measurement of the findings. The absence of any declaration on allocation concealment (just one study mentioned it) and the randomization process (stated in five studies) is the most critical issue. The Cochrane Risk of Bias Tool 2 has a dose of subjectivity in the assessment, and we deemed most of the studies to have some concerns of bias. However, if there had been no allocation concealment, in reality, the trials would have been regarded as having a high risk of bias overall. This is more troublesome for parallel designs, although they only accounted for roughly a third of the total in our assessment. Nevertheless, we performed subgroup analyses for studies with high bias and some concerns of bias and the main results of our review remained statistically significant in both cases. Another disadvantage is the relatively small number of individuals per research; nevertheless, systematic inclusion of a large number of publications helps to increase overall power. We had a long list of potential outcomes, but only a few papers provided measurements for several of them. For some outcomes, there was an important heterogeneity, but after the sensitivity leave-one-out analysis, they seemed robust and remained statistically significant.

Additionally, our review has several strengths: (1) the publications’ methodological flaws were assessed using the newest edition of the Cochrane Collaboration’s Risk of Bias Tool, version 2, one of the most prestigious organizations that conducts systematic reviews and creates high-quality instruments for study validity evaluation; (2) a comprehensive search strategy was used; (3) many databases (PubMed, Embase, Scopus, Cochrane Database) were searched; (4) only randomized controlled trials were included; (5) sensitivity and subgroup analyses were performed; and (6) twenty-two metabolic syndrome and inflammatory markers in middle-aged and older adults were assessed.

Future studies should focus more on inflammatory markers that were assessed in only a small number of studies, but with significant results. The value close to statistical significance level of fasting blood glucose suggests a need for further studies to check if this was a spurious result or a real useful signal. Furthermore, the quality of future randomized controlled trials on walnut diets should be improved, especially regarding allocation concealment, the randomization process, and intention to treat analyses.

## 5. Conclusions

In conclusion, despite some heterogeneity in the intervention outcomes, our meta-analysis found significant amelioration in the lipid profiles (TG, TC, and LDL-C levels) with walnut consumption compared with different control diets in the studied age category, middle-aged and older adults. Incipient data from a single study [47], which should be further investigated, suggest that long-term walnut consumption displayed potential benefits in lowering inflammation and indirectly on preventing several age-related diseases. Even though further and better-designed studies are needed to strengthen these findings, the results stress the importance of including walnuts in the dietary plans of middle-aged and older populations.

## Figures and Tables

**Table 1:** Characteristics of the selected studies.

Reference	Country	Study (RCT) Design	Exposure Period	Washout Period	Participants (*n*), Health Status	Age (Years), (SD)/(IQR) (Range)	Female (%)	Walnut Intervention (g/d)	Control Intervention	Outcome of Interest
Zambόn et al., 2000 [31]	Spain, USA	Crossover	6 weeks	0	49 polygenic hypercholesterolemia *	56 (±11)	47%	41–56 g/d (18% of the energy need)	MedD (no walnut)	BW, TC, LDL-C, HDL-C, TG
Ros et al., 2004 [32]	Spain	Crossover	4 weeks	0	20 healthy, non-smokers (hypercholesterolemia)	55 (±55.9)	60%	40–65 g/d (18% of energy need)	MedD (no walnut)	BW, SBP, DBP, TC, LDL-C, HDL-C, CRP,
Tapsell et al., 2004 [33]	Australia	Parallel	6 months	NA	58 T2D *	59.3 (±8.1)	41.37%	30 g/d—walnut-enriched modified low-fat diet	Modified low-fat diet (no walnuts)	BW, BMI, HbA1c, TC, LDL-C, HDL-C, TG
Olmedilla- Alonso et al., 2008 [34]	Spain	Crossover	5 weeks	1 month	25 CV risk, smokers	54.4 (±8.1)	40%	19.4 g/d (20% walnut-enriched meat products)	Restructured meat products (no walnut)	TC, HDL-C, LDL-C, TG, BW, SBP, DBP
Spaccarotella et al., 2008 [35]	USA	Crossover	8 weeks	2 weeks	21 healthy, non-smokers	65.9 (55–75)	0%	75 g/d (24% of energy need)	Western-type diet (no walnut)	SBP, DBP, TC, HDL-C, LDL-C
Tapsell et al., 2009 [36]	Australia	Parallel	1 year	NA	50 T2D *	54 (±8.7)	NI	30 g/d (walnut-enriched 2000 kcal diet, 30% fat)	2000 kcal diet, 30% fat (no walnut)	BW, FBG, TC, HDL-C, LDL-C, TG, HbA1c, insulin
Ma et al., 2010 [37]	USA	Crossover	8 weeks	8 weeks	21 T2D, non-smokers	58.1 (±9.2)	58.30%	56 g/d	Habitual diet (no walnut)	TC, HDL-C, LDL-C, TG, FPG, insulin, HOMA-IR, BW, BMI, WC, SBP, DBP
Torabian et al., 2010 [38]	USA	Crossover	6 months	0	87 healthy, non-smokers	54 (±10.2)	56%	28–64 g/d (12% of energy need)	Habitual diet (no walnut)	TC, LDL-C, HDL-C, TG
Canales et al., 2011 [39]	Spain	Crossover	5 weeks	4–6 weeks	22 CV risk, smokers	54.8 (±9.4)	40%	34–29 g/d (20% walnut-enriched meat)	Low-fat meat products (no walnut)	VCAM-1, ICAM-1, HDL-C
Katz et al., 2012 [40]	USA	Crossover	8 weeks	4 weeks	40 healthy, non-smokers (overweight, MetS risk)	57.4 (±11.9)	60.9%	56 g/d	Habitual diet (no walnut)	TC, HDL-C, LDL-C, TG, FPG, insulin, HOMA-IR, BW, BMI, WC, SBP, DBP
Wu et al., 2014 [41]	Germany, USA	Crossover	8 weeks	2 weeks	40 healthy *	60 (±6.32)	75%	43 g/d (replacing 30 g saturated fat in Western-type diet)	Western-type diet (no walnut)	TC, LDL-C, HDL-C, FBG, insulin, HOMA-IR, HbA1c, VCAM-1, ICAM-1
Bamberger et al., 2017 [42]	Germany	Crossover	8 weeks	4 weeks	194 healthy, non-smokers	63 (±7)	69%	43 g/d	Western-type diet (no walnut)	TC, LDL-C, HDL-C, TG
Bitok et al., 2018 [43]	USA, Spain	Parallel	2 years	NA	307 healthy *	69.4 (±3.9)	67%	28; 42; 56 g/d (15% of energy need)	Habitual diet (no walnut)	BW, WC
Domènech et al., 2019 [44]	USA, Spain	Parallel	2 years	NA	236 healthy * (60% mild hyper-tension)	68.8 (±3.3)	65%	30–60 g/d, (15% of energy need)	Habitual diet (no walnut)	SBP, DBP
Sanchis et al., 2019 [45]	Spain	Crossover	30 days	30 days	13 CKD *	71 (±10.11)	46.20%	30 g/d (walnut-enriched CKD diet)	CKD patients’ diet (no walnut)	BMI, TC, HDL-C, LDL-C, TG, FBG, HbA1c, CRP
Abdrabalnabi et al., 2020 [46]	USA, Spain	Parallel	2 years	NA	625 healthy *	69.1 (±3.6)	67%	30; 45; 60 g/d (15% of energy need)	Habitual diet (no walnut)	BMI, SBP, DBP, TG, HDL-C, FBG
Cofán et al., 2020 [47]	USA, Spain	Parallel	2 years	NA	634 healthy *	69.1 (±3.6)	66%	30; 45; 60 g/d (15% of energy need)	Western-type diet (no walnut)	VCAM-1, ICAM-1, IL-6, IFN-γ, IL-1β, TNF-α, E-selectin, hs-CRP

*—non-specified smoking status; RCT—randomized controlled trials; NA—not applicable; BMI—body mass index; BW—body weight; CKD—chronic kidney disease; CV—cardiovascular; CRP—C-reactive protein; hs-CRP—high-sensitivity C-reactive protein; DBP—diastolic blood pressure; FBG—fasting blood glucose; HbA1c—glycosylated hemoglobin A1c; HDL-C—high-density lipoprotein cholesterol; HOMA-IR—homeostatic model assessment for insulin resistance; ICAM—intercellular adhesion molecule; IFN-γ—interferon gamma; IL-1β—interleukin-1β; IL-6—interleukin-6; IQR—interquartile range; LDL-C—low-density lipoprotein cholesterol; MedD—Mediterranean diet; MetS—metabolic syndrome; NI—no information; SBP—systolic blood pressure; SD—standard deviation; T2D—type 2 diabetes; TC—total cholesterol; TG—triglycerides; TNF-α—tumor necrosis factor-alpha; VCAM—the vascular cell adhesion molecule; WC—waist circumference.

**Table 2:** Effects of walnut-enriched diets on inflammatory and metabolic syndrome biomarkers.

Characteristic, Effect Size Type, SMD	Effect Size (95% CI)	*p*-Value	I^2^ (95% CI)	*p*-Value	Egger Test	Studies
CRP (mg/L)	−0.37 (−1.39–0.65)	0.478	NC		NC	[32,45]
hs-CRP (mg/L)	−0.01 (−0.12–0.11)	0.903	NC		NC	[47]
IFN-γ (pg/mL)	−1.26 (−2.01–−0.51)	<0.001	NC		NC	[47]
IL-6 (pg/mL)	−0.18 (−0.33–−0.03)	0.021	NC		NC	[47]
IL-1β (pg/mL)	−0.1 (−0.16–−0.04)	<0.001	NC		NC	[47]
TNF-α (pg/mL)	−0.31 (−0.54–−0.08)	0.009	NC		NC	[47]
E-selectin (ng/mL)	−2.57 (−4.09–−1.05)	<0.001	NC		NC	[47]
ICAM-1 (ng/mL)	−0.02 (−0.11–0.07) ANC	0.672	-	-	-	[39,41,47]
VCAM-1 (ng/mL)	−0.11 (−0.32–0.1) ANC	0.305	-	-	-	[39,41,47]
WC (cm)	−0.14 (−0.8–0.51)	0.671	0 (0–89.6)	0.71	0.572	[37,40,43]
BMI (kg/m^2^)	0.11 (−0.11–0.34)	0.326	63.1 (2.4–86)	0.028	0.683	[33,37,40,45,46]
BW (kg)	0 (−0.4–0.39)	0.987	22.2 (0–64.1)	0.253	0.537	[31,32,33,34,36,37,40,43]
SBP (mmHg)	−0.85 (−4.48–2.77)	0.644	64.4 (24–83.4)	0.006	0.699	[32,34,35,37,40,44,45,46]
DBP (mmHg)	−0.34 (−1.68–1)	0.62	35.3 (0–71.4)	0.146	0.551	[32,34,35,37,40,44,45,46]
FBG (mg/dL)	0.01 (0–0.02)	0.088	0 (0–74.6)	0.692	0.57	[36,37,40,41,45,46]
TG (mg/dL)	−7.41 (−10.89–−3.94)	<0.001	99.1 (99–99.3)	<0.001	0.264	[31,32,33,34,35,36,37,38,40,41,42,45,46]
TC (mg/dL)	−5.22 (−7.64–−2.8)	<0.001	97.4 (96.5–98.1)	<0.001	0.375	[31,32,34,35,36,37,38,40,41,42,45]
HDL-C (mg/dL)	−0.18 (−0.59–0.22)	0.375	47.4 (0–72.4)	0.029	0.507	[31,32,33,34,35,36,37,38,39,40,41,42,45,46]
LDL-C (mg/dL)	−5.93 (−7.77–−4.09)	<0.001	24.8 (0–61.8)	0.2	0.83	[31,32,33,34,35,36,37,38,40,41,42,45]
HbA1c (%)	0.08 (−0.04–0.2)	0.196	0 (0–84.7)	0.774	0.816	[33,36,41,45]
HOMA-IR	0.03 (−0.44–0.5)	0.891	57.1 (0–87.8)	0.097	0.95	[37,40,41]
Insulin (mIU/mL)	0.91 (−2.16–3.98)	0.561	65.4 (0–88.2)	0.034	0.505	[36,37,40,41]

ANC—algorithm did not converge (when study [39] was entered; thus, the result is based only on studies [41,47]); BMI—body mass index; BW—body weight; CI—confidence interval; CRP—C-reactive protein; hs-CRP—high-sensitivity C-reactive protein; DBP—diastolic blood pressure; FBG—fasting blood glucose; HbA1c—glycosylated hemoglobin A1c; HDL-C—high-density lipoprotein cholesterol; HOMA-IR—homeostatic model assessment for insulin resistance; ICAM—intercellular adhesion molecule; IFN-γ—interferon gamma; IL-1β—interleukin-1β; IL-6—interleukin-6; LDL-C—low-density lipoprotein cholesterol; NC—not computed for less than three studies; SBP—systolic blood pressure; SD—standard deviation; SMD—standardized mean change difference; TC—total cholesterol; TG—triglycerides; TNF-α—tumor necrosis factor-alpha; VCAM—vascular cell adhesion molecule; WC—waist circumference.

## Supplementary Materials

The following supporting information can be downloaded at: https://www.mdpi.com/article/10.3390/antiox11071412/s1.

## Abbreviations

ALA	α-linolenic acid
apoB	apolipoprotein B
BMI	body mass index
BW	body weight
CI	confidence interval
CI	confidence interval
CRP	C-reactive protein
CVD	cardiovascular diseases
DBP	diastolic blood pressure
eTE	the standard error of the treatment effect
FBG	fasting blood glucose
HbA1c	glycosylated hemoglobin A1c
HDL-C	high density lipoprotein-cholesterol
HOMA-IR	homeostatic model assessment for insulin resistance
hs-CRP	high-sensitivity C-reactive protein
ICAM-1	intercellular adhesion molecule-1
IF	interferon gamma
IL-1β	interleukin-1β
IL-6	interleukin-6
IQR	interquartile ranges
LDL-C	low density lipoprotein-cholesterol
MedD	Mediterranean diet
MetS	metabolic syndrome
MUFAs	monounsaturated fatty acids
NF-кB	nuclear factor kappa B
Nrf2/ARE	nuclear factor erythroid 2-related factor 2/antioxidant response element
PUFAs	polyunsaturated fatty acids
RCT	randomized controlled trial
ROS	reactive oxygen species
SBP	systolic blood pressure
SD	standard deviation
SE	standard error
SMD	standardized mean difference
SMD	standardized mean change difference
T2D	type 2 diabetes
TC	total cholesterol
TE	treatment effect
TG	triglycerides
TNF-α	tumor necrosis factor-alpha
VCAM-1	vascular cell adhesion molecule-1
W	weight
WC	waist circumference
