# Supplementary material for: Walnut Intake Interventions Targeting Biomarkers of Metabolic Syndrome and Inflammation in Middle-Aged and Older Adults: A Systematic Review and Meta-Analysis of Randomized Controlled Trials
Source: Antioxidants (Basel). 2022 Jul 21;11(7):1412. doi: 10.3390/antiox11071412 (PMC9312161; doi:10.3390/antiox11071412)
Supplement: Supplementary file 1 [file antioxidants-11-01412-s001.zip › antioxidants-1789298-supplementary.pdf]

Supplementary file

Walnut Intake Interventions Targeting Biomarkers of Metabolic Syndrome and Inflammation in Middle-aged and Older Adults: A Systematic Review and Meta-Analysis of Randomized Controlled Trials

Antioxidants

Letiția Mateș [1], Daniela-Saveta Popa [1,*], Marius Emil Rusu [2,*], Ionel Fizeșan [1], Daniel Leucuța [3]

[1] Department of Toxicology, Faculty of Pharmacy, Iuliu Hatieganu University of Medicine and Pharmacy, 8 Victor Babes, 400012 Cluj-Napoca, Romania

[2] Department of Pharmaceutical Technology and Biopharmaceutics, Faculty of Pharmacy, Iuliu Hatieganu University of Medicine and Pharmacy, 8 Victor Babes, 400012 Cluj-Napoca, Romania

[3] Department of Medical Informatics and Biostatistics, Faculty of Medicine, Iuliu Hatieganu University of Medicine and Pharmacy, 8 Victor Babes, 400012 Cluj-Napoca, Romania

* Correspondence: dpopa@umfcluj.ro (D.-S.P.); rusu.marius@umfcluj.ro (M.E.R.); Tel.: +40-264-450-555 (D.-S.P.)

Table S1. Search strategies for each database.

**Pubmed**
("juglans"[MeSH Terms] OR "juglans"[All Fields] OR "walnut"[All Fields] OR "walnuts"[All Fields])<br>AND<br>(<br>("waist circumference"[MeSH Terms] OR ("waist"[All Fields] AND "circumference"[All Fields]) OR "waist circumference"[All Fields] OR<br>"obesity, abdominal"[MeSH Terms] OR ("obesity"[All Fields] AND "abdominal"[All Fields]) OR "abdominal obesity"[All Fields] OR<br>("central"[All Fields] AND "obesity"[All Fields]) OR "central obesity"[All Fields] OR<br>"obesity"[MeSH Terms] OR "obesity"[All Fields] OR "obese"[All Fields] OR "obeses"[All Fields] OR "obesities"[All Fields] OR<br>"overweight"[MeSH Terms] OR "overweight"[All Fields] OR<br>"body mass index"[MeSH Terms] OR ("body"[All Fields] AND "mass"[All Fields] AND "index"[All Fields]) OR "body mass index"[All Fields] OR "BMI"[Title/Abstract] OR<br>"body weight"[MeSH Terms] OR ("body"[All Fields] AND "weight"[All Fields]) OR "body weight"[All Fields] OR<br>"kilogram"[All Fields] OR "kilogramme"[All Fields] OR "kilograms"[All Fields] OR<br>"kg"[Title/Abstract] OR "lbs"[Title/Abstract] OR "pound"[All Fields] OR "pounds"[All Fields])<br>OR

("blood pressure"[MeSH Terms] OR "blood pressure determination"[MeSH Terms] OR "arterial pressure"[MeSH Terms] OR ("blood"[All Fields] AND "pressure"[All Fields]) OR "blood pressure"[All Fields] OR "arterial pressure"[MeSH Terms] OR ("arterial"[All Fields] AND "pressure"[All Fields]) OR "arterial pressure"[All Fields] OR "SBP"[Title/Abstract] OR "DBP"[Title/Abstract] OR<br>"hypertension"[MeSH Terms] OR "hypertension"[All Fields] OR "hypertensions"[All Fields] OR "hypertensive"[All Fields] OR "hypertensives"[All Fields] OR<br>"blood glucose"[MeSH Terms] OR "glucose"[MeSH Terms] OR "glucose"[All Fields] OR ("blood"[All Fields] AND "glucose"[All Fields]) OR "glycemia"[All Fields] OR "glycemias"[All Fields] OR<br>"diabetes mellitus"[MeSH Terms] OR "diabete"[All Fields] OR ("diabetes"[All Fields] AND "mellitus"[All Fields]) OR "diabetes mellitus"[All Fields] OR "diabetic"[All Fields] OR "diabetics"[All Fields] OR<br>"triglycerides"[MeSH Terms] OR "triglycerid"[All Fields] OR "triglycerides"[All Fields] OR "triglyceride"[All Fields] OR "triglycerids"[All Fields] OR<br>"cholesterol"[MeSH Terms] OR "cholesterol"[All Fields] OR "HDL"[Title/Abstract] OR "LDL"[Title/Abstract])<br>OR<br>("c reactive protein"[MeSH Terms] OR ("c reactive"[All Fields] AND "protein"[All Fields]) OR "c reactive protein"[All Fields] OR "crp"[Title/Abstract] OR<br>"interferons"[MeSH Terms] OR "interferone"[All Fields] OR "interferones"[All Fields] OR "IFN"[Title/Abstract] OR<br>"interleukins"[MeSH Terms] OR "interleukine"[All Fields] OR "interleukines"[All Fields] OR "IL"[Title/Abstract] OR<br>("e selectin"[MeSH Terms] OR "e selectin"[All Fields]<br>"cell adhesion molecules"[MeSH Terms] OR ("cell"[All Fields] AND "adhesion"[All Fields] AND "molecules"[All Fields]) OR "cell adhesion molecules"[All Fields] OR ("intercellular"[All Fields] AND "adhesion"[All Fields] AND "molecule"[All Fields]) OR "intercellular adhesion molecule"[All Fields]) OR "CAM"[Title/Abstract] OR "ICAM"[Title/Abstract] OR<br>("vascular cell adhesion molecule 1"[MeSH Terms] OR ("vascular"[All Fields] AND "cell"[All Fields] AND "adhesion"[All Fields] AND "molecule 1"[All Fields]) OR "vascular cell adhesion molecule 1"[All Fields] OR ("vascular"[All Fields] AND "cell"[All Fields] AND "adhesion"[All Fields] AND "molecule"[All Fields]) OR "vascular cell adhesion molecule"[All Fields]) OR "VCAM"[Title/Abstract] OR "sVCAM"[Title/Abstract] OR<br>"tumor necrosis factor alpha"[MeSH Terms] OR "tumour necrosis factor"[All Fields] OR ("tumor"[All Fields] AND "necrosis"[All Fields] AND "factor alpha"[All Fields]) OR "tumor necrosis factor alpha"[All Fields] OR ("tumor"[All Fields] AND "necrosis"[All Fields] AND "factor"[All Fields]) OR "TNF"[Title/Abstract])<br>)<br>AND<br>((randomized controlled trial [pt] OR controlled clinical trial [pt] OR randomized [tiab] OR placebo [tiab] OR clinical trials as topic [mesh: noexp] OR randomly [tiab] OR trial [ti]) NOT (animals [mh] NOT humans [mh]))
**EMBASE**
('juglans'/exp OR 'walnut'/exp OR 'walnut':ti,ab OR 'walnuts':ti,ab) AND ('metabolic' AND ('syndrome'/exp OR 'syndrome') OR 'metabolic syndrome'/exp OR 'metabolic syndrome' OR (((('waist'/exp OR waist) AND ('circumference'/exp OR circumference) OR 'obesity, abdominal'/exp OR (abdominal AND ('obesity'/exp OR obesity)) OR 'central'/exp OR central) AND ('obesity'/exp OR obesity) OR 'obesity'/exp OR obesity OR obese OR 'overweight'/exp OR overweight OR (('body'/exp OR body) AND ('mass'/exp OR mass) AND ('index'/exp OR index)) OR 'bmi'/exp OR 'bmi':ti,ab OR 'body'/exp OR body) AND ('weight'/exp OR weight)) OR kilogram OR 'kg' OR 'lbs'

OR pounds OR (('blood'/exp OR blood) AND ('pressure'/exp OR pressure)) OR 'hypertension'/exp OR hypertension OR 'sbp':ti,ab OR 'dbp':ti,ab OR (('blood'/exp OR blood) AND ('glucose'/exp OR glucose)) OR 'glucose'/exp OR glucose OR 'glycemia'/exp OR glycemia OR 'diabetes'/exp OR diabetes OR 'triglycerides'/exp OR triglycerides OR 'cholesterol'/exp OR cholesterol OR 'hdl'/exp OR 'hdl':ti,ab OR 'ldl'/exp OR 'ldl':ti,ab OR ('c reactive protein'/exp OR 'c reactive protein' OR 'CRP':ti,ab OR<br>'interferons'/exp OR 'interferone'/exp OR 'interferone' OR 'interferones' OR 'IFN':ti,ab OR 'interleukins'/exp OR 'interleukine' OR 'interleukines' OR 'interleukins' OR 'IL':ti,ab OR<br>'e selectin'/exp OR 'e selectin' OR<br>'cell adhesion molecules'/exp OR 'cell adhesion molecules' OR ('cell' AND 'adhesion' AND 'molecules') OR 'intercellular adhesion molecule'/exp OR 'intercellular adhesion molecule' OR ('intercellular' AND 'adhesion' AND 'molecules') OR 'CAM':ti,ab OR 'ICAM':ti,ab OR 'sICAM':ti,ab OR<br>'vascular cell adhesion molecule 1'/exp OR ('vascular' AND 'cell' AND 'adhesion' AND 'molecule') OR 'vascular cell adhesion molecule'/exp OR 'vascular cell adhesion molecule' OR 'VCAM':ti,ab OR 'sVCAM':ti,ab OR<br>'tumour necrosis factor'/exp OR 'tumor necrosis factor alpha'/exp OR ('tumor' AND 'necrosis' AND ('factor' OR 'factor alpha')) OR 'TNF':ti,ab)) AND (('randomized controlled trial'/exp OR 'controlled clinical study'/exp OR random$:ti,ab OR 'randomization'/exp OR 'intermethod comparison'/exp OR placebo:ti,ab OR compare:ti OR compared:ti OR comparison:ti OR ((evaluated:ab OR evaluate:ab OR evaluating:ab OR assessed:ab OR assess:ab) AND (compare:ab OR compared:ab OR comparing:ab OR comparison:ab)) OR (open:ti,ab AND adj:ti,ab AND label:ti,ab) OR ((double:ti,ab OR single:ti,ab OR doubly:ti,ab OR singly:ti,ab) AND adj:ti,ab AND (blind:ti,ab OR blinded:ti,ab OR blindly:ti,ab)) OR 'double blind procedure'/exp OR parallel) AND group$1:ti,ab OR crossover:ti,ab OR 'cross over':ti,ab OR ((assign$:ti,ab OR match:ti,ab OR matched:ti,ab OR allocation:ti,ab) AND adj5:ti,ab AND (alternate:ti,ab OR group$1:ti,ab OR intervention$1:ti,ab OR patient$1:ti,ab OR subject$1:ti,ab OR participant$1:ti,ab)) OR assigned:ti,ab OR allocated:ti,ab OR (controlled:ti,ab AND adj7:ti,ab AND (study:ti,ab OR design:ti,ab OR trial:ti,ab)) OR volunteer:ti,ab OR volunteers:ti,ab OR 'human experiment'/exp OR trial:ti) NOT ((((((random$:ti,ab AND adj:ti,ab AND sampl$:ti,ab AND adj7:ti,ab AND ('cross section$':ti,ab OR questionnaire$1:ti,ab OR survey$:ti,ab OR database$1:ti,ab) NOT ('comparative study'/exp OR 'controlled study'/exp OR 'randomi?ed controlled':ti,ab OR 'randomly assigned':ti,ab) OR 'cross-sectional study'/exp) NOT ('randomized controlled trial'/exp OR 'controlled clinical study'/exp OR 'controlled study'/exp OR 'randomi?ed controlled':ti,ab OR 'control group$1':ti,ab) OR (case:ti,ab AND adj:ti,ab AND control$:ti,ab AND random$:ti,ab NOT 'randomi?ed controlled':ti,ab) OR ('systematic review':ti NOT (trial:ti OR study:ti)) OR (nonrandom$:ti,ab NOT random$:ti,ab) OR 'random field$':ti,ab OR ('random cluster':ti,ab AND adj3:ti,ab AND sampl$:ti,ab) OR (review:ab AND review:pt)) NOT trial:ti OR 'we searched':ab) AND (review:ti OR review:pt) OR 'update review':ab OR (databases:ab AND adj4:ab AND searched:ab) OR rat:ti OR rats:ti OR mouse:ti OR mice:ti OR swine:ti OR porcine:ti OR murine:ti OR sheep:ti OR lambs:ti OR pigs:ti OR piglets:ti OR rabbit:ti OR rabbits:ti OR cat:ti OR cats:ti OR dog:ti OR dogs:ti OR cattle:ti OR bovine:ti OR monkey:ti OR monkeys:ti OR trout:ti OR marmoset$1:ti) AND 'animal experiment'/exp OR 'animal experiment'/exp) NOT ('human experiment'/exp OR 'human'/exp))
**Scopus**
TITLE-ABS-KEY("juglans" OR "juglans" OR "walnut" OR "walnuts")<br>AND<br>TITLE-ABS-KEY(<br>("waist circumference" OR ("waist" AND "circumference") OR<br>("obesity" AND "abdominal") OR "abdominal obesity" OR<br>("central" AND "obesity") OR "central obesity" OR<br>"obesity" OR "obesity" OR "obese" OR "obeses" OR "obesities" OR

"overweight" OR "overweight" OR<br>"body mass index" OR ("body" AND "mass" AND "index") OR ("BMI") OR<br>"body weight" OR ("body" AND "weight") OR<br>"kilogram" OR "kilogramme" OR "kilograms" OR ("kg") OR ("lbs") OR "pound" OR "pounds")<br>OR<br>("blood pressure" OR "blood pressure determination" OR "arterial pressure" OR ("blood" AND "pressure") OR "arterial pressure" OR ("arterial" AND "pressure") OR ("SBP") OR ("DBP") OR<br>"hypertension" OR "hypertension" OR "hypertensions" OR "hypertensive" OR "hypertensives" OR<br>"blood glucose" OR "glucose" OR ("blood" AND "glucose") OR "glycemia" OR "glycemias" OR<br>"diabetes mellitus" OR "diabete" OR ("diabetes" AND "mellitus") OR "diabetes mellitus" OR<br>"diabetic" OR "diabetics" OR<br>"triglycerides" OR "triglycerid" OR "triglyceride" OR "triglycerids" OR<br>"cholesterol" OR ("HDL") OR ("LDL"))<br>OR<br>("c reactive protein" OR ("c reactive" AND "protein") OR ("crp") OR<br>"interferons" OR "interferone" OR "interferones" OR ("IFN") OR<br>"interleukins" OR "interleukine" OR "interleukines" OR ("IL") OR<br>("e selectin" OR "e selectin"<br>"cell adhesion molecules" OR ("cell" AND "adhesion" AND "molecules") OR ("intercellular" AND "adhesion" AND "molecule") OR "intercellular adhesion molecule") OR ("CAM") OR ("ICAM") OR<br>("vascular cell adhesion molecule 1" OR ("vascular" AND "cell" AND "adhesion" AND "molecule 1") OR ("vascular" AND "cell" AND "adhesion" AND "molecule") OR "vascular cell adhesion molecule") OR ("VCAM") OR ("sVCAM") OR<br>"tumor necrosis factor alpha" OR "tumour necrosis factor" OR ("tumor" AND "necrosis" AND "factor alpha") OR ("tumor" AND "necrosis" AND "factor") OR ("TNF"))<br>)<br>AND<br>("randomized controlled trial" OR ("randomized" AND "controlled" AND "trial") OR TITLE-ABS-KEY(randomized) OR TITLE-ABS-KEY(placebo) OR ("clinical" AND ("trial" OR "trials")) OR TITLE-ABS-KEY(randomly) OR TITLE(trial)) AND NOT (TITLE-ABS-KEY(rat) OR TITLE-ABS-KEY(rats) OR TITLE-ABS-KEY(mouse) OR TITLE-ABS-KEY(mice) OR TITLE-ABS-KEY(swine) OR TITLE-ABS-KEY(porcine) OR TITLE-ABS-KEY(murine) OR TITLE-ABS-KEY(sheep) OR TITLE-ABS-KEY(lambs) OR TITLE-ABS-KEY(pigs) OR TITLE-ABS-KEY(piglets) OR TITLE-ABS-KEY(rabbit) OR TITLE-ABS-KEY(rabbits) OR TITLE-ABS-KEY(cat) OR TITLE-ABS-KEY(cats) OR TITLE-ABS-KEY(dog) OR TITLE-ABS-KEY(dogs) OR TITLE-ABS-KEY(cattle) OR TITLE-ABS-KEY(bovine) OR TITLE-ABS-KEY(monkey) OR TITLE-ABS-KEY(monkeys) OR TITLE-ABS-KEY(trout))<br>AND (LIMIT-TO (DOCTYPE , "ar") OR LIMIT-TO (DOCTYPE , "re") OR LIMIT-TO (DOCTYPE , "le")) AND (LIMIT-TO (SUBJAREA , "MEDI")) AND (LIMIT-TO (SRCTYPE , "j")) AND (LIMIT-TO (EXACTKEYWORD , "Human") OR LIMIT-TO (EXACTKEYWORD , "Humans"))
**Cochrane**
("juglans" OR "juglans" OR "walnut" OR "walnuts")<br>AND<br>(<br>("waist circumference" OR ("waist" AND "circumference") OR<br>("obesity" AND "abdominal") OR "abdominal obesity" OR<br>("central" AND "obesity") OR "central obesity" OR<br>"obesity" OR "obesity" OR "obese" OR "obeses" OR "obesities" OR<br>"overweight" OR "overweight" OR<br>"body mass index" OR ("body" AND "mass" AND "index") OR ("BMI") OR<br>"body weight" OR ("body" AND "weight") OR

"kilogram" OR "kilogramme" OR "kilograms" OR ("kg") OR ("lbs") OR "pound" OR "pounds")<br>OR<br>("blood pressure" OR "blood pressure determination" OR "arterial pressure" OR ("blood" AND "pressure") OR "arterial pressure" OR ("arterial" AND "pressure") OR ("SBP") OR ("DBP") OR "hypertension" OR "hypertension" OR "hypertensions" OR "hypertensive" OR "hypertensives" OR "blood glucose" OR "glucose" OR ("blood" AND "glucose") OR "glycemia" OR "glycemias" OR "diabetes mellitus" OR "diabete" OR ("diabetes" AND "mellitus") OR "diabetes mellitus" OR "diabetic" OR "diabetics" OR<br>"triglycerides" OR "triglycerid" OR "triglyceride" OR "triglycerids" OR<br>"cholesterol" OR ("HDL") OR ("LDL"))<br>OR<br>("c reactive protein" OR ("c reactive" AND "protein") OR ("crp") OR<br>"interferons" OR "interferone" OR "interferones" OR ("IFN") OR<br>"interleukins" OR "interleukine" OR "interleukines" OR ("IL") OR<br>("e selectin" OR "e selectin"<br>"cell adhesion molecules" OR ("cell" AND "adhesion" AND "molecules") OR ("intercellular" AND "adhesion" AND "molecule") OR "intercellular adhesion molecule") OR ("CAM") OR ("ICAM") OR ("vascular cell adhesion molecule 1" OR ("vascular" AND "cell" AND "adhesion" AND "molecule 1") OR ("vascular" AND "cell" AND "adhesion" AND "molecule") OR "vascular cell adhesion molecule") OR ("VCAM") OR ("sVCAM") OR<br>"tumor necrosis factor alpha" OR "tumour necrosis factor" OR ("tumor" AND "necrosis" AND "factor alpha") OR ("tumor" AND "necrosis" AND "factor") OR ("TNF"))<br>)
**ClinicalTrials**
intervention: ("juglans" OR "juglans" OR "walnut" OR "walnuts")

Table S2. Search strategies for each database.

Reference	Exclusion criteria
**Zambón et al. 2000** [31]	Persons whose elevated blood cholesterol levels had a strong genetic basis (such as heterozygous familial hypercholesterolemia or familial combined hyperlipidemia)
**Ros et al. 2004** [32]	Not-mentioned
**Tapsell et al. 2004** [33]	Insulin therapy / HbA1c >9%; BMI > 35 kg/m^2 with major debilitating illness; Known food allergies or food habits inhibiting the study; Illiteracy; Inadequate conversational English
**Olmedilla-Alonso at al. 2008** [34]	The use of vitamin or mineral supplements, hormone replacement therapy, regular use of aspirin, medications known to affect lipid absorption or metabolism and any chronic disease (i.e. diabetes); Individuals with blood pressure over 145/ 95 mm Hg or taking medication for hypertension were initially excluded, but, given the small number of subjects willing to participate, it was necessary to include patients being treated for this condition (n = 4, taking enalaprilate, lacidipine or candesartan)
**Spaccarotella et al. 2008** [35]	Allergies to nuts and use of prescription and non-prescription preparations known to alter PSA (e.g. Saw Palmetto, Finesteride), hormone levels, blood pressure or blood lipids;

	Men taking vitamin E supplements were eligible if they discontinued use two months before entering the study
**Tapsell et al. 2009** [36]	Major illnesses; food allergies or inhibitory habits, illiteracy and/or inadequate English
**Ma et al. 2010** [37]	Current use of vasoactive medications or supplements, current eating disorder, known atherosclerotic vascular disease, sleep apnea; Pregnancy; Restricted diet; Allergy to walnuts or other nuts; Use of lipid lowering or antihypertensive medications unless stable condition with medication for at least 3 months; Willingness to refrain from taking medication for 12 h before assessment
**Torabian et al. 2010** [38]	Not-mentioned
**Canales et al. 2011** [39]	Familiar hypercholesterolemia and/or type I diabetes; Lipid-lowering, anti-hypertensive, anti-inflammatory treatments or hormone therapy; Volunteers who did not frequently consume meat (≥5 times/week)
**Katz et al. 2012** [40]	Pregnancy; Participants diagnosed with atherosclerotic vascular disease, diabetes, severe hypertension, sleep apnea, tuberculosis, acquired immune deficiency syndrome, cancer, psychotic disorder, and/or eating disorder; Participants with a prior history of substance abuse, consumed restricted diets by choice (i.e., vegan, carbohydrate-restricted, etc.); Allergic to walnuts or any other nuts, or unwilling to refrain from taking medication for 12 hours prior to assessment; Regularly used non-steroidal anti-inflammatory drugs or vasoactive medication, fiber supplements, aspirin, lipid-lowering medications, or antihypertensive medications and had been taking them at a stable dosage for less than 3 months
**Wu et al. 2014** [41]	BMI ≥ 35 kg/m^2, LDL-C ≥ 190 mg/dL, TG ≥ 350 mg/dL; Acute or chronic inflammation; Acute malignancy; Uncontrolled thyroid disease or other endocrine diseases, any systemic disease (e.g. hypertension, diabetes mellitus, liver or kidney disease); Known nut allergy or lactose intolerance; Tobacco, drug or alcohol abuse (women: > 70 g/week, men: > 140 g/week) or treatment with antidiabetic, hypolipidemic, antihypertensive or anti-inflammatory drugs, vitamin E or hormonal replacement therapy in the previous 3 months
**Bamberger et al. 2017** [42]	Persons with a history of cardiovascular and atherosclerotic disease, a known allergy to tree nuts, a vegan or ovo-lacto vegetarian lifestyle, and patients on regular medication (except stable treatment of thyroid disease and hypertension)
**Bitok et al. 2018** [43]	Inability to undergo neuropsychological testing; Morbid obesity: BMI ≥ 40 kg/m^2; Uncontrolled diabetes: HbA1c > 8%; Uncontrolled HTA: on-treatment BP ≥ 150/100 mmHg; Prior stroke, significant head trauma or brain surgery; Relevant psychiatric illness; Major depression; Cognitive deterioration or dementia with a score < 24 on the Mini-Mental State Examination (MMSE); Other neurodegenerative disorders like

	Parkinson's disease; advanced AMD or eye-related conditions precluding ophthalmological evaluation; Prior chemotherapy; Chronic illness with projected shortened lifespan; Allergy to walnuts; Customary use of fish oil and/or tree nuts (> 2 servings/week) and/or other relevant sources of ALA, such as flaxseed oil or soy lecithin
**Domènech et al 2019** [44]	Morbid obesity: BMI ≥40 kg/m²; Uncontrolled diabetes: HbA1c >8%; Uncontrolled HTA (on treatment BP ≥150/100 mmHg); Prior stroke or major head trauma; Any relevant psychiatric illness, advanced cognitive deterioration (mild cognitive impairment or frank dementia), other neurodegenerative diseases (ie, Parkinson disease); Any chronic illness expected to shorten survival; Bereavement; Allergy to walnuts; Customary use of fish oil, flaxseed oil, or soy lecithin supplements
**Sanchis et al. 2019** [45]	Not-mentioned
**Abdrabalnabi et al. 2020** [46]	Extreme obesity: BMI ≥ 40 kg/m²; Uncontrolled diabetes or hypertension; Allergy to walnuts.
**Cofán et al. 2020** [47]	Not-mentioned

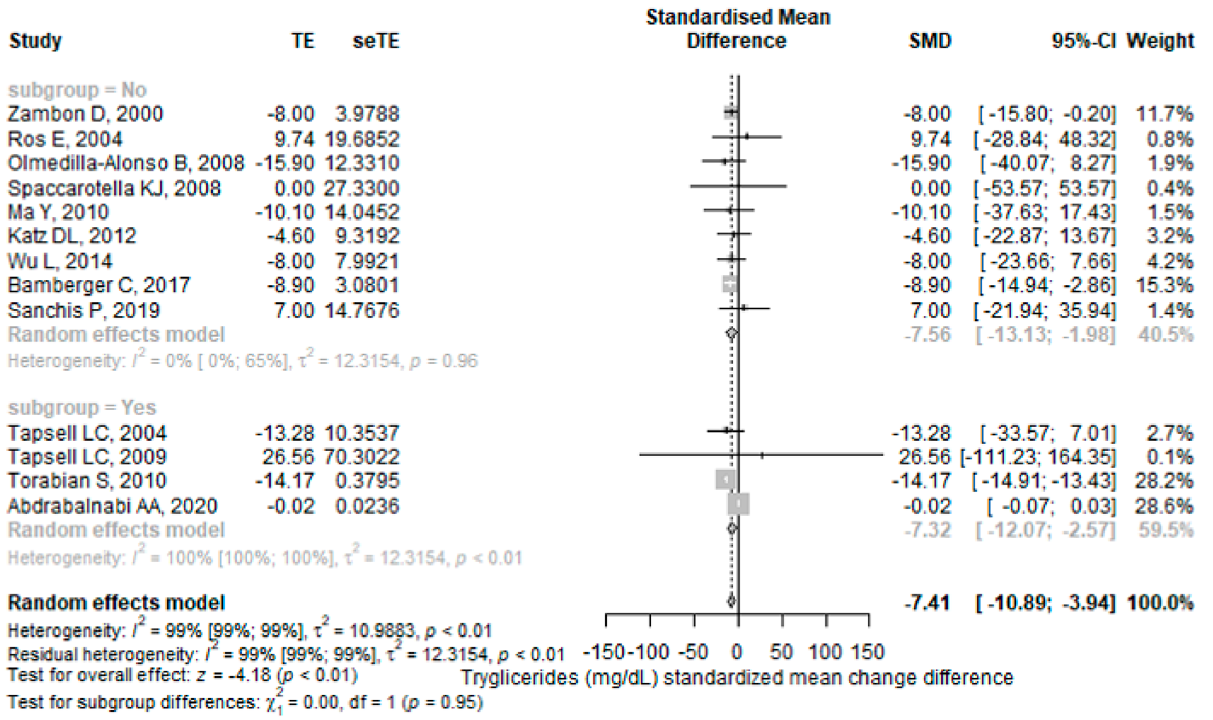

Figure S1. Forest plot for **TG** (mg/dL) standardized mean change difference, comparing with subgroup analyses for Exposure duration ≥ 8 weeks.

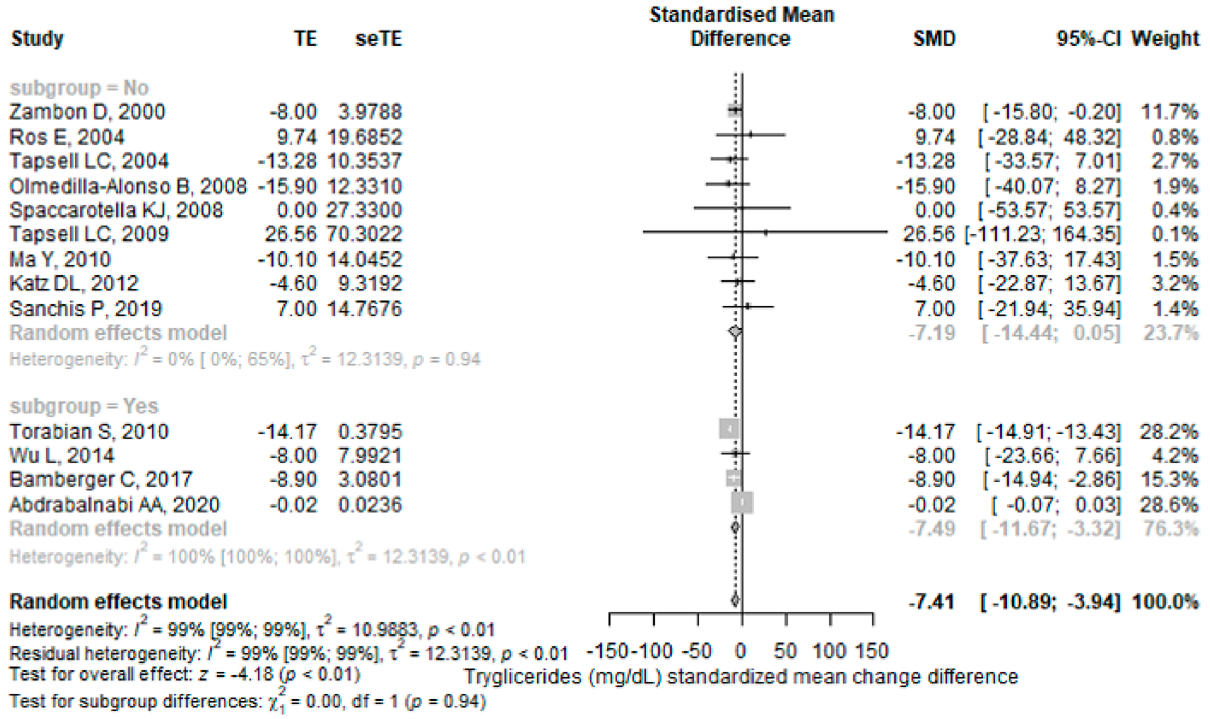

Figure S2. Forest plot for **TG** (mg/dL) standardized mean change difference, comparing with subgroup analyses for Healthy.

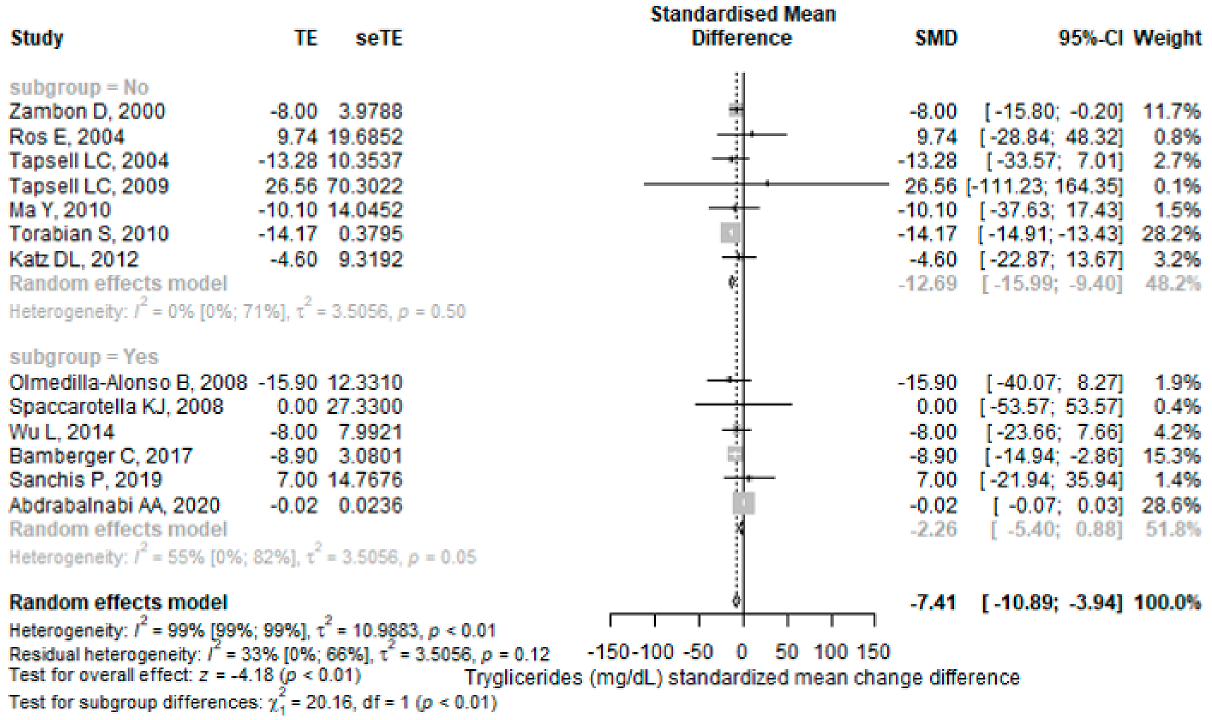

Figure S3. Forest plot for **TG** (mg/dL) standardized mean change difference, comparing with subgroup analyses for 40 years of age or mean age ≥ 50 years.

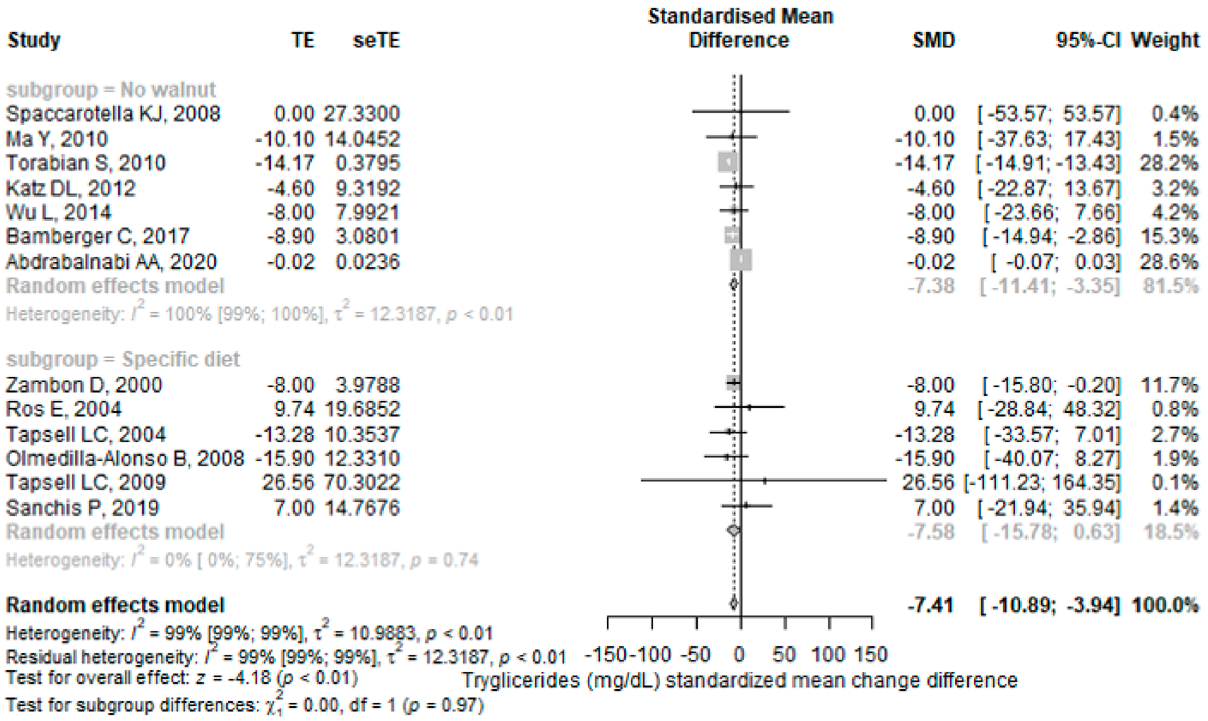

Figure S4. Forest plot for **TG** (mg/dL) standardized mean change difference, comparing with subgroup analyses for Control.

....

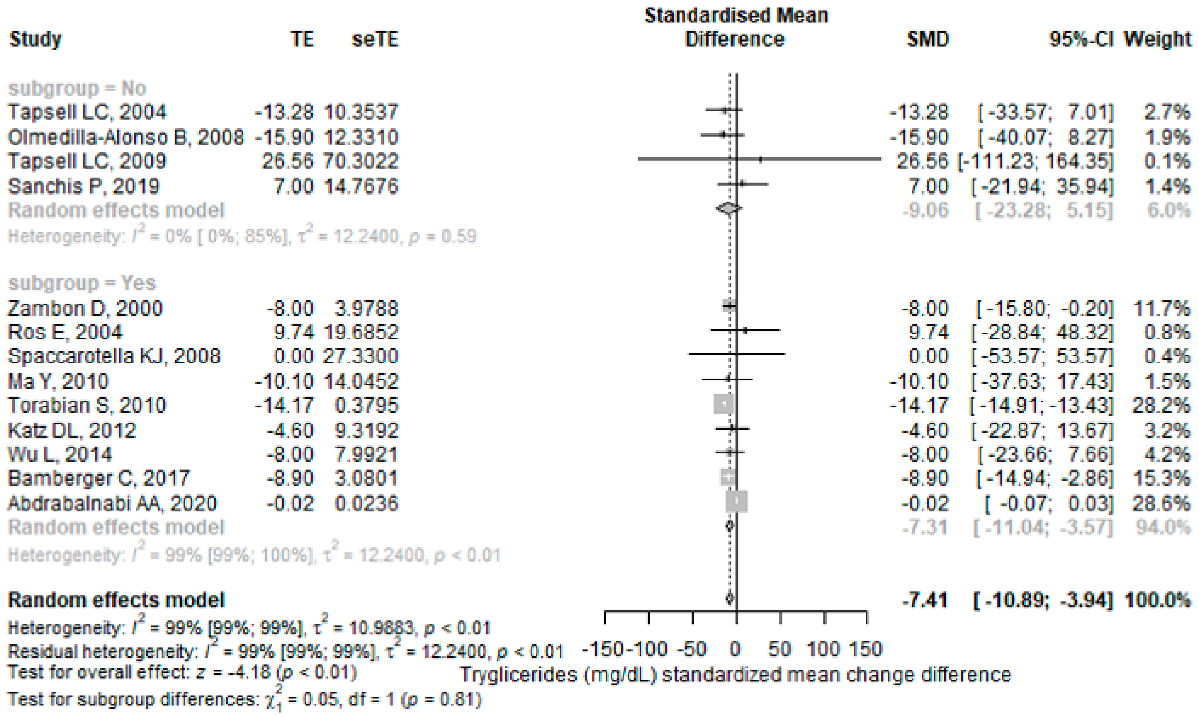

Figure S5. Forest plot for **TG** (mg/dL) standardized mean change difference, comparing with subgroup analyses for Walnut ≥ 42 g/day.

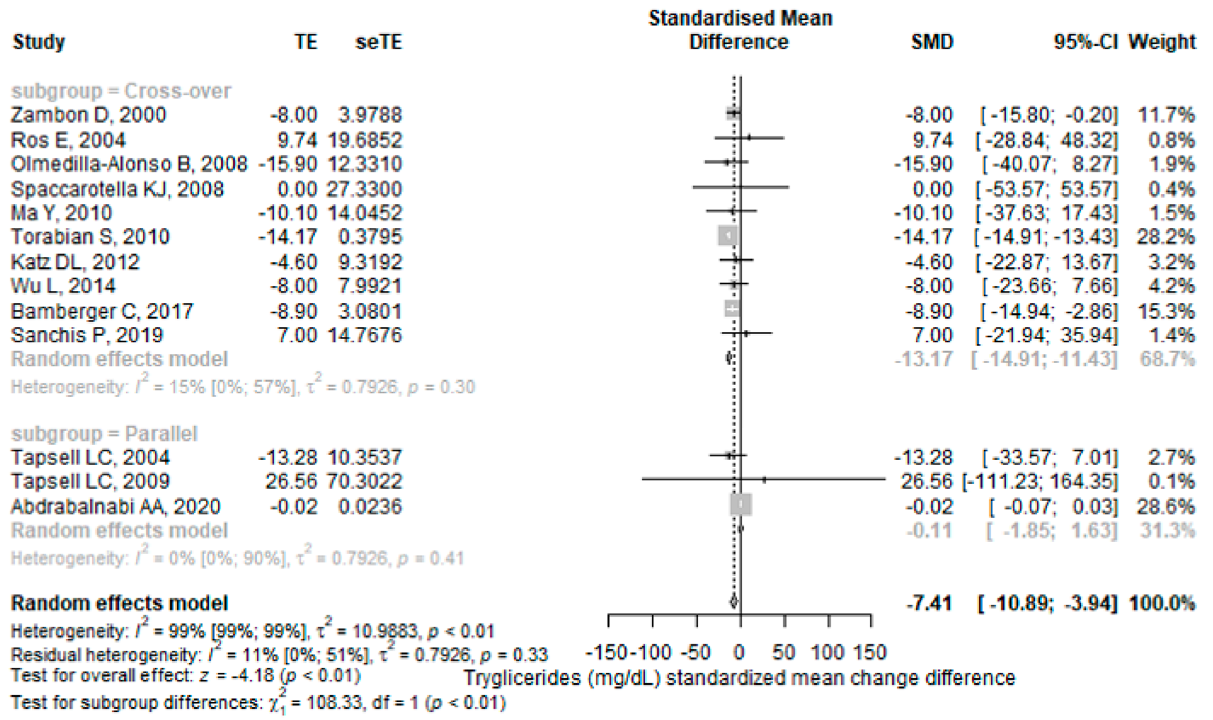

Figure S6. Forest plot for **TG** (mg/dL) standardized mean change difference, comparing with subgroup analyses for Trial design.

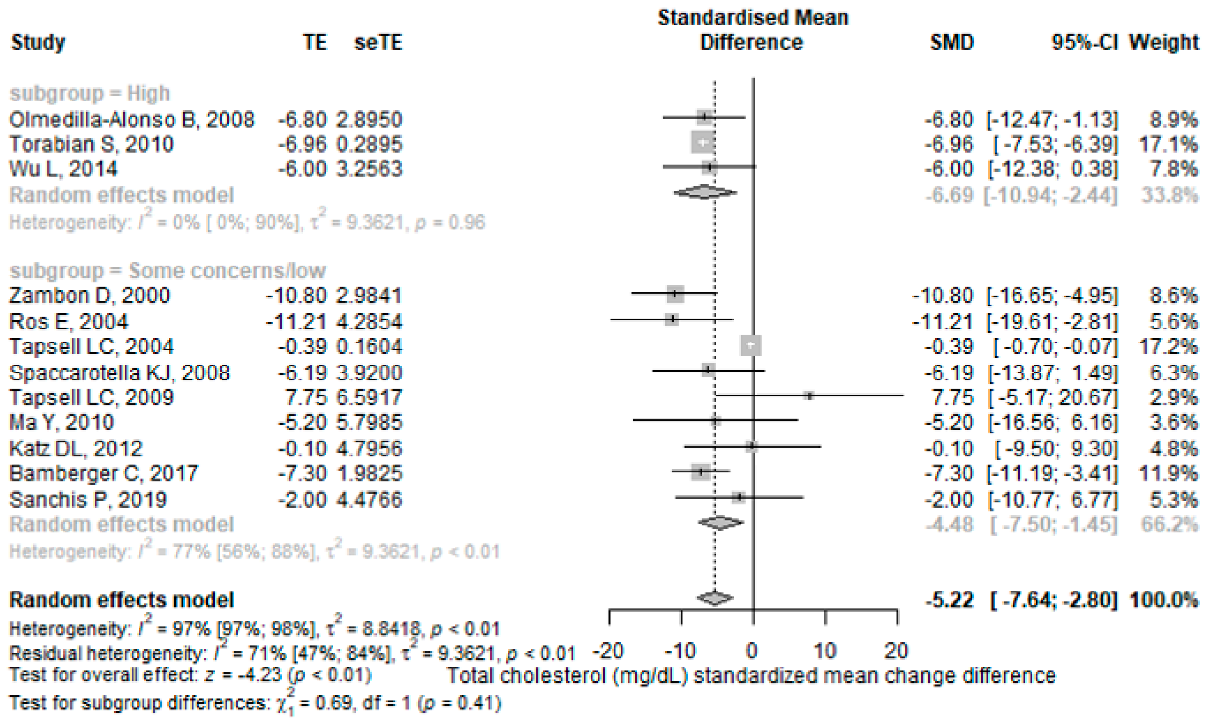

Figure S7. Forest plot for **TC** (mg/dL) standardized mean change difference, comparing with subgroup analyses for Risk of bias.

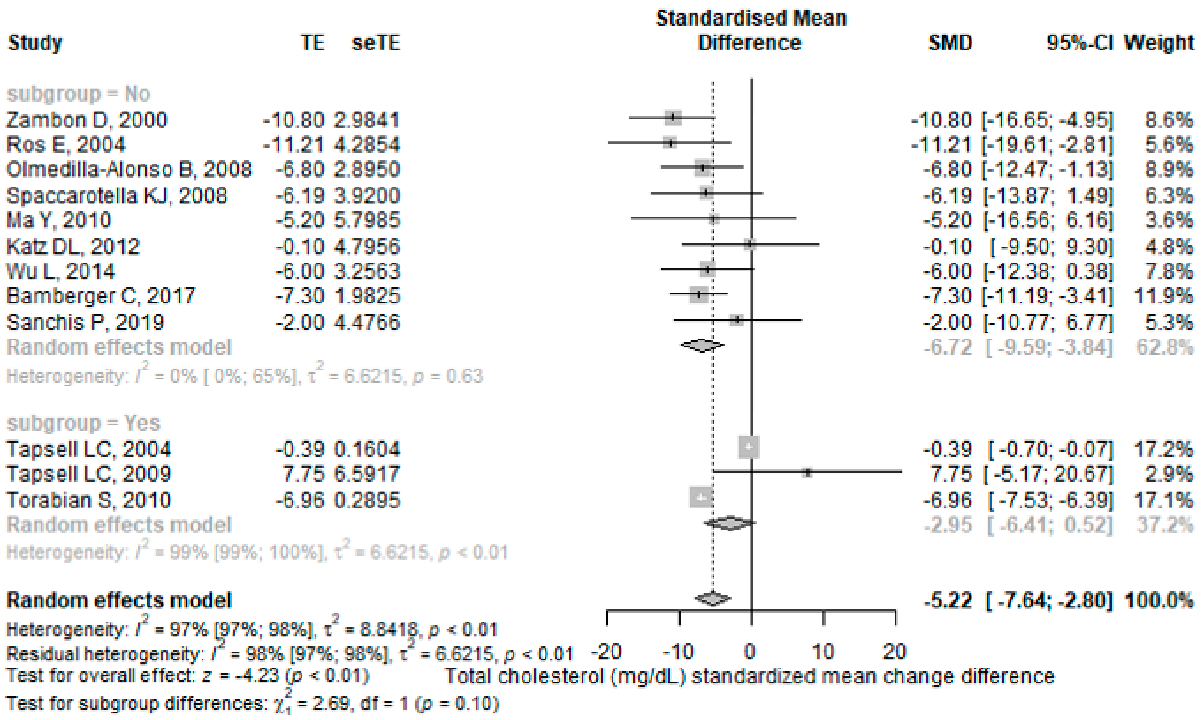

Figure S8. Forest plot for **TC** (mg/dL) standardized mean change difference, comparing with subgroup analyses for Exposure duration ≥ 8 weeks.

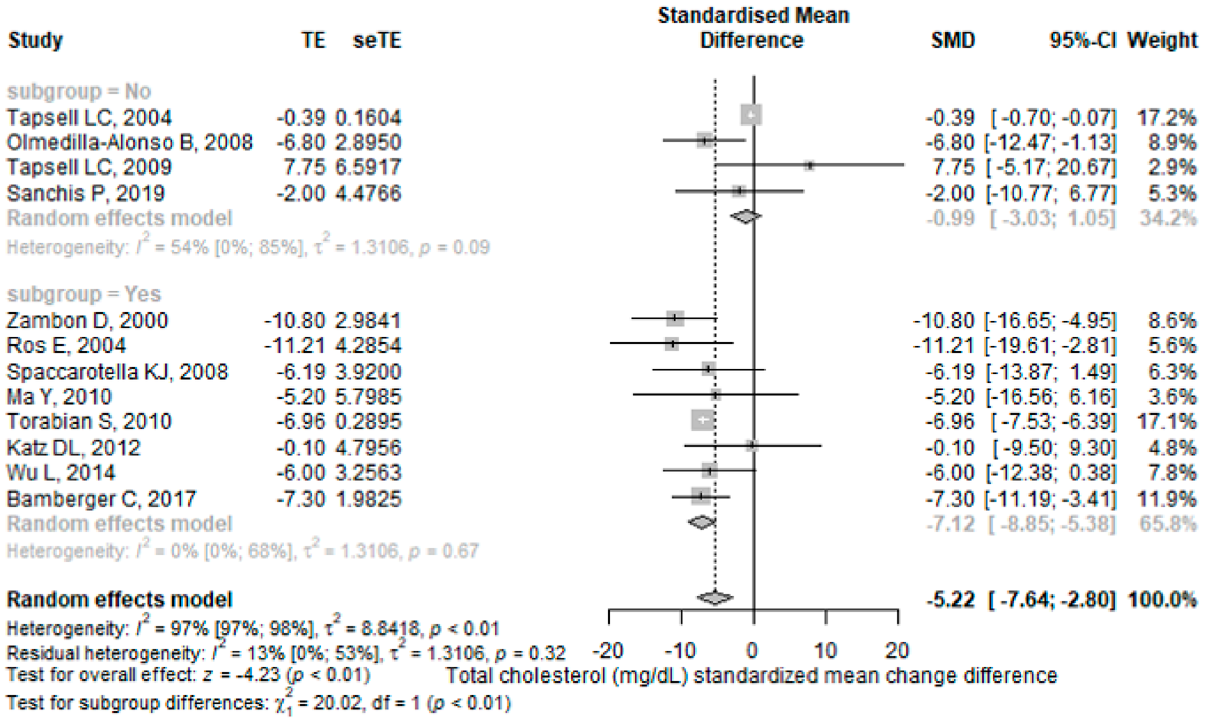

Figure S9. Forest plot for **TC** (mg/dL) standardized mean change difference, comparing with subgroup analyses for Walnut ≥ 42 g/day.

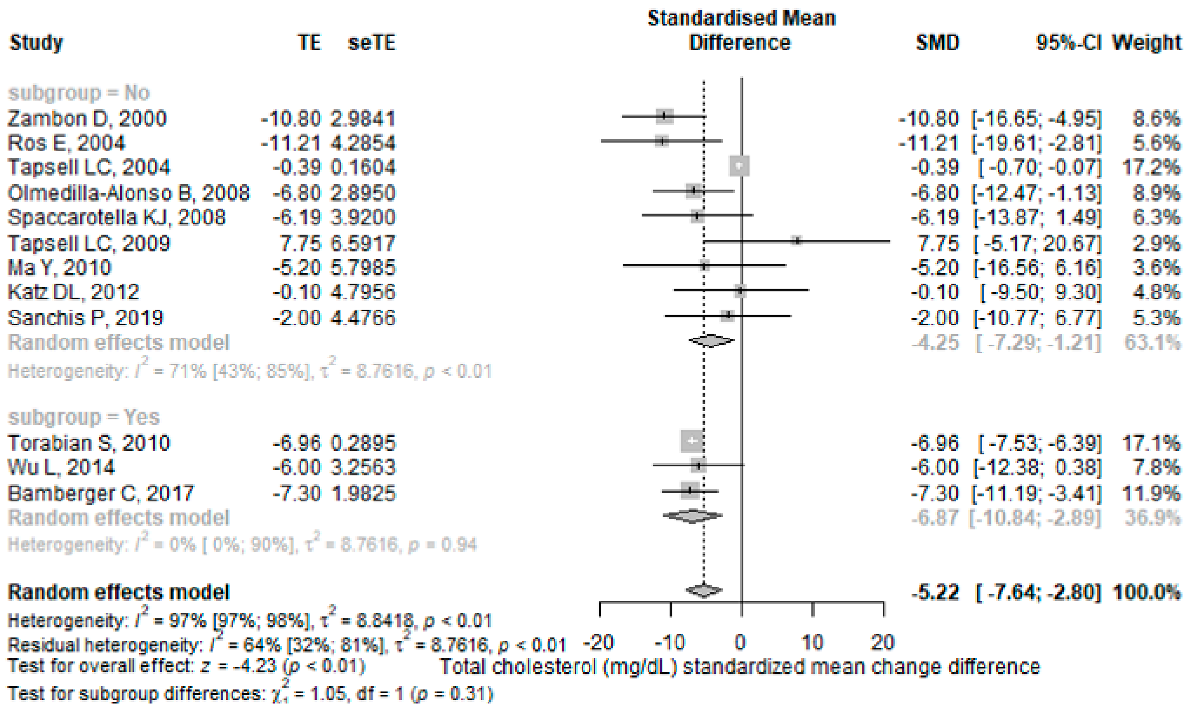

Figure S10. Forest plot for **TC** (mg/dL) standardized mean change difference, comparing with , with subgroup analyses for Healthy.

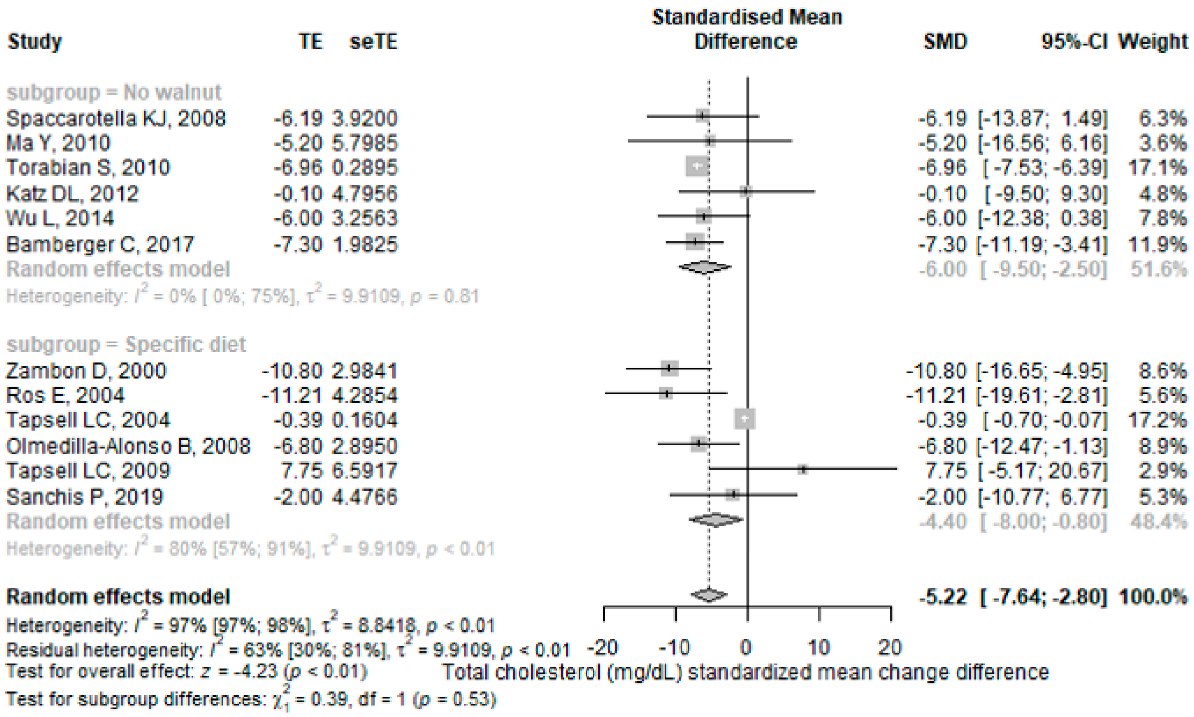

Figure S11. Forest plot for **TC** (mg/dL) standardized mean change difference, comparing with subgroup analyses for Control.

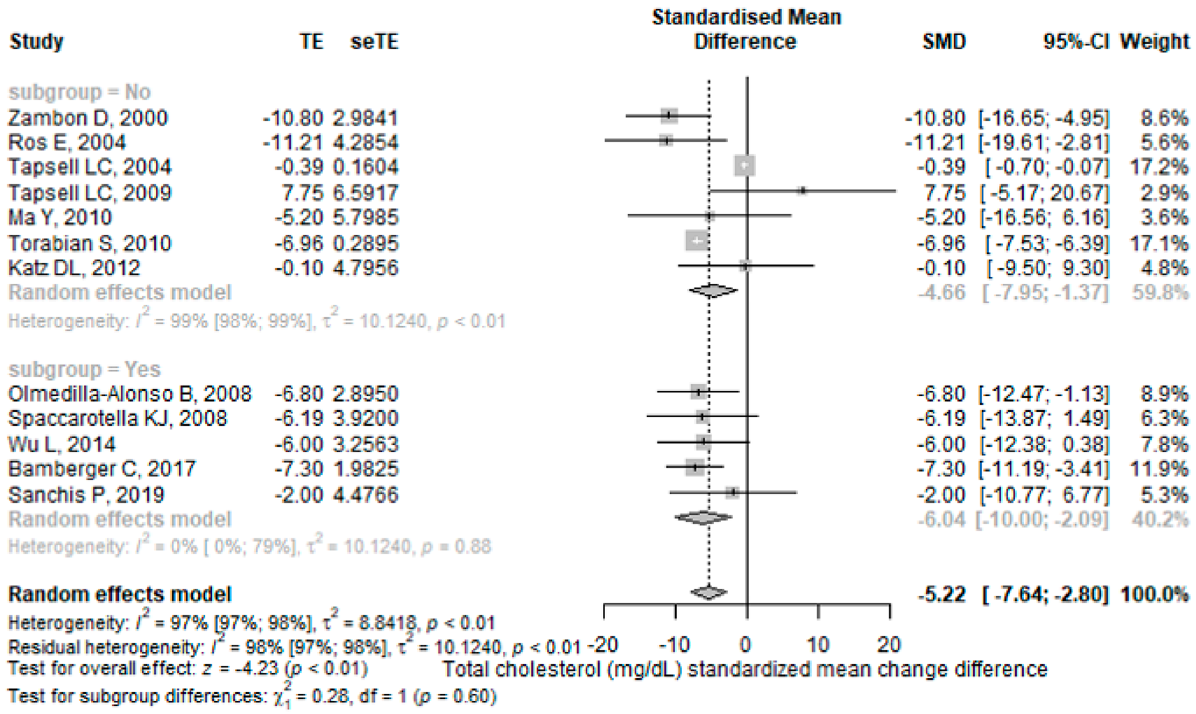

Figure S12. Forest plot for **TC** (mg/dL) standardized mean change difference, with subgroup analyses for 40 years of age or mean age ≥ 50 years.

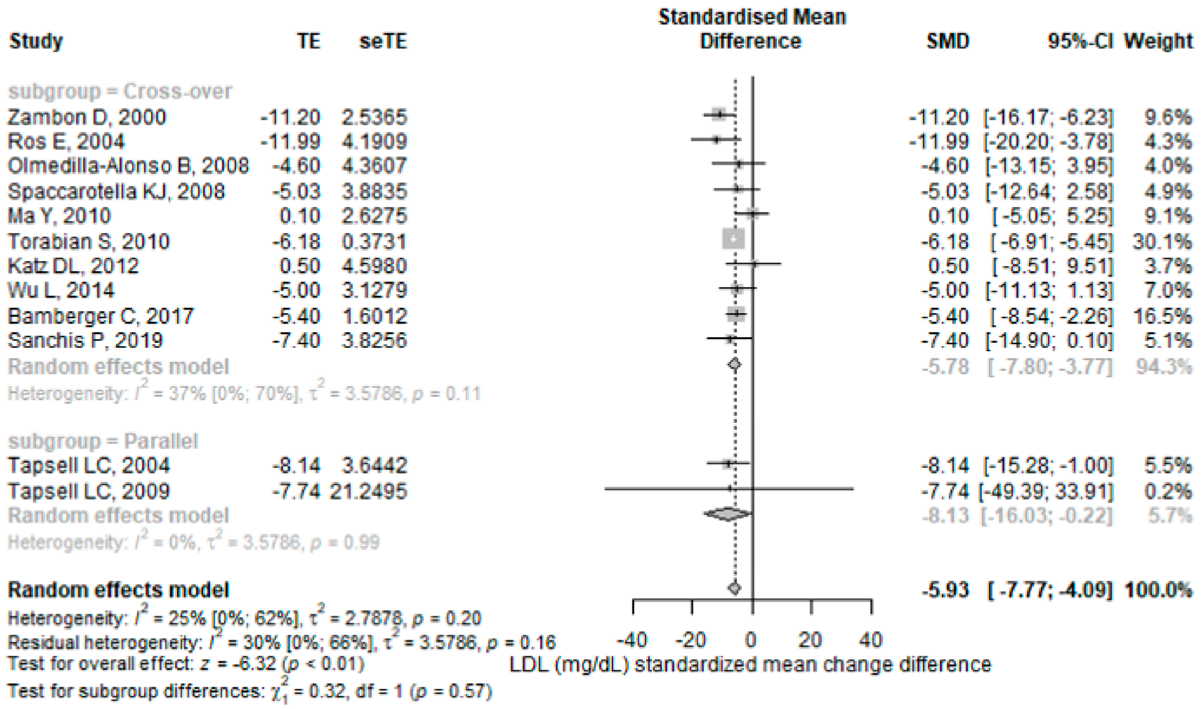

Figure S13. Forest plot for **LDL-C** (mg/dL) standardized mean change difference, comparing with subgroup analyses for Trial design.

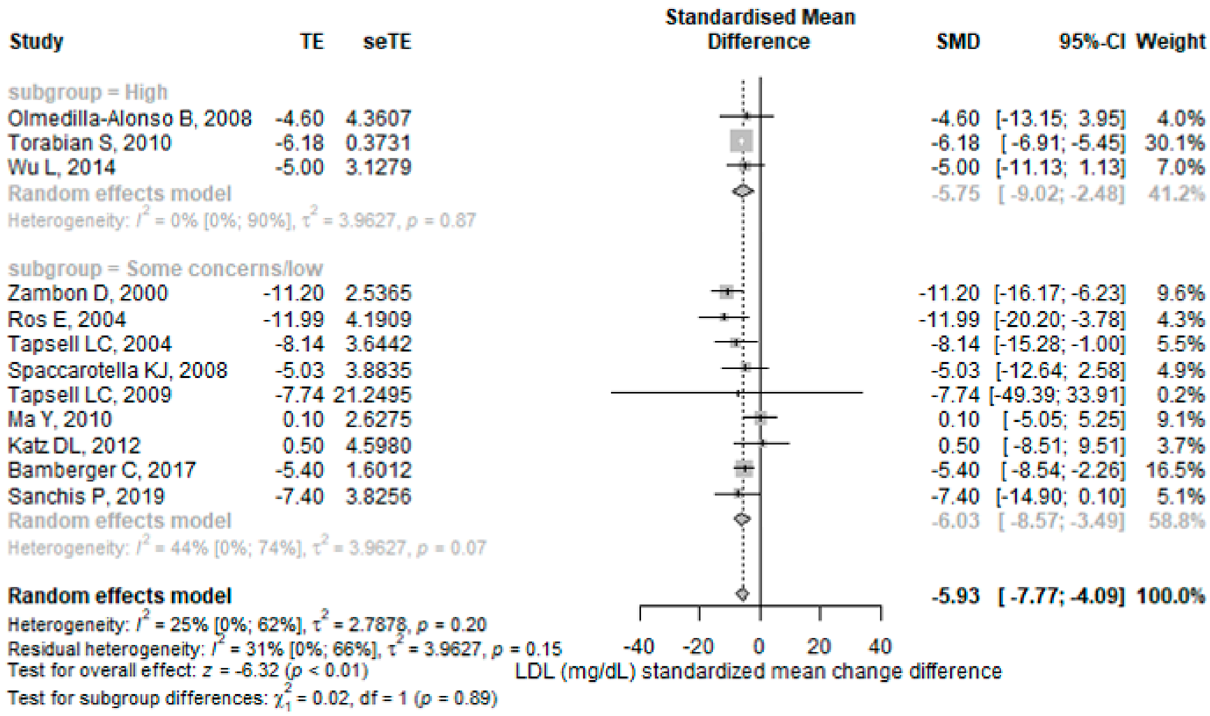

Figure S14. Forest plot for **LDL-C** (mg/dL) standardized mean change difference, comparing with subgroup analyses for Risk of bias.

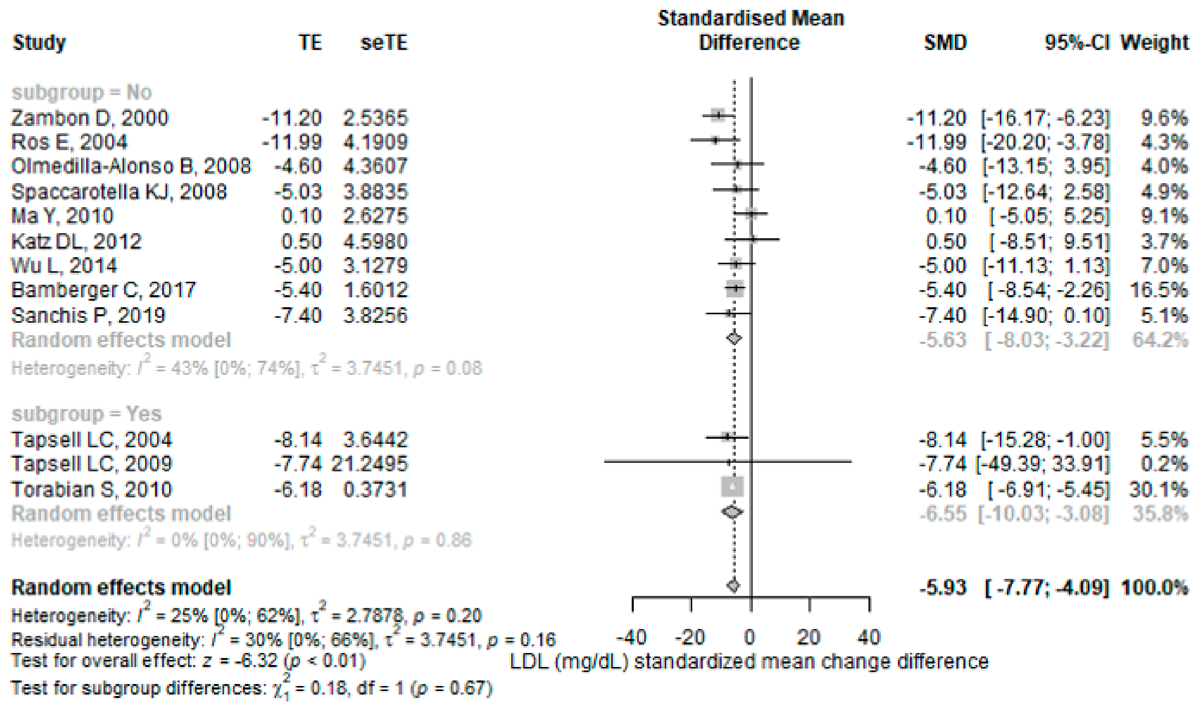

Figure S15. Forest plot for **LDL-C** (mg/dL) standardized mean change difference, comparing with subgroup analyses for Exposure duration ≥ 8 weeks.

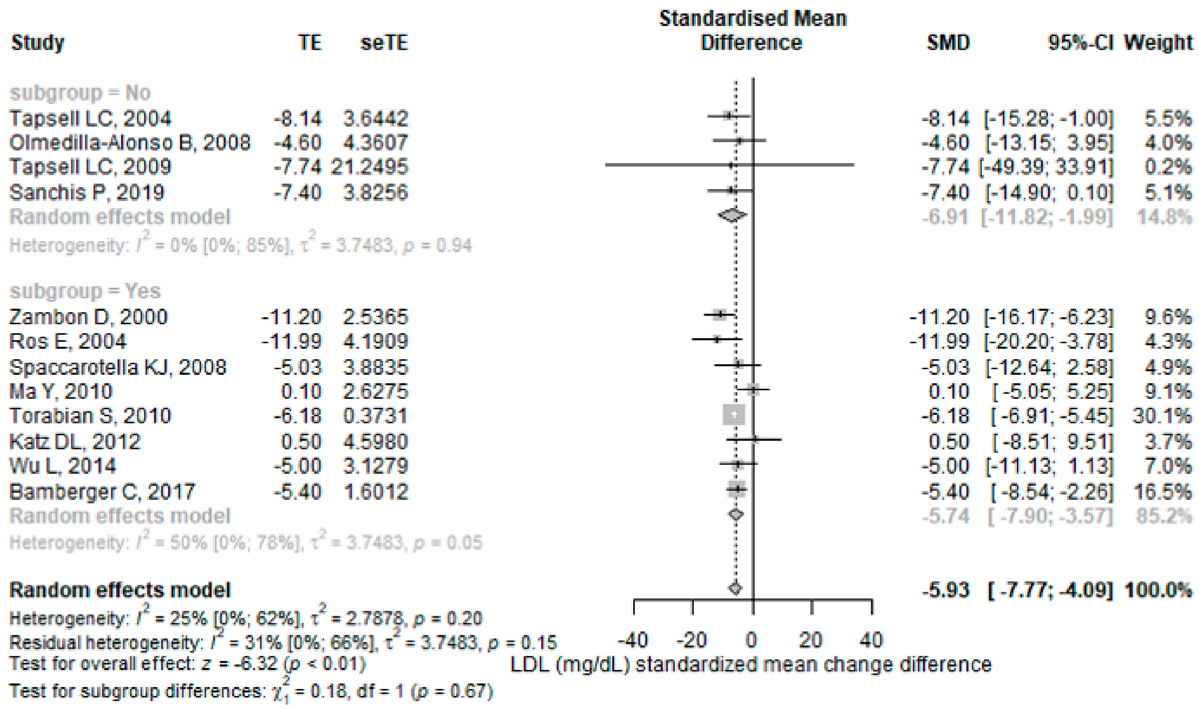

Figure S16. Forest plot for **LDL-C** (mg/dL) standardized mean change difference, comparing with subgroup analyses for Walnut ≥ 42 g/day.

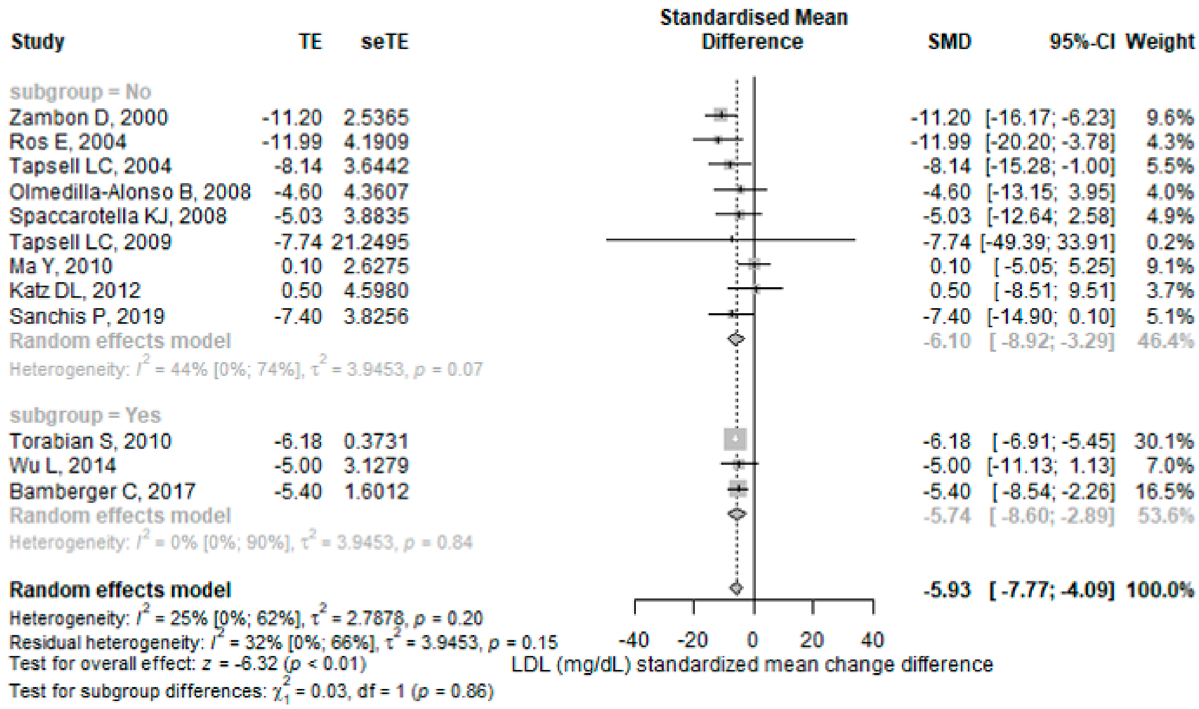

Figure S17. Forest plot for **LDL-C** (mg/dL) standardized mean change difference, comparing with subgroup analyses for Healthy.

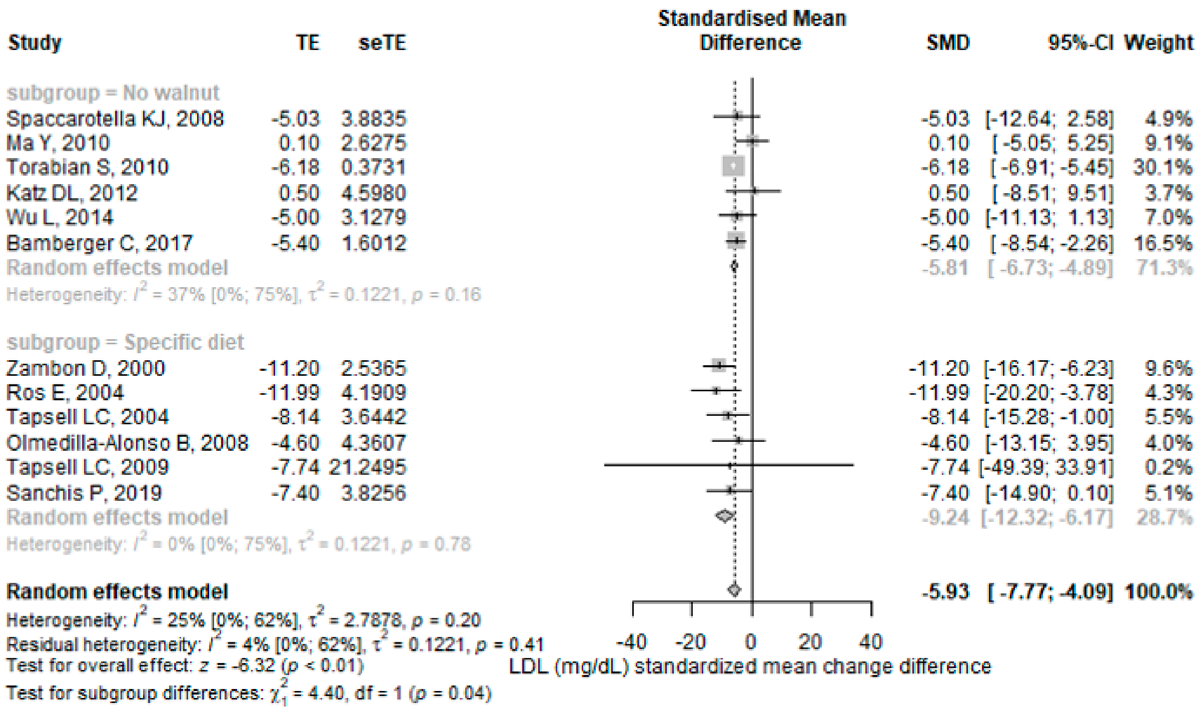

Figure S18. Forest plot for **LDL-C** (mg/dL) standardized mean change difference, comparing with subgroup analyses for Control.

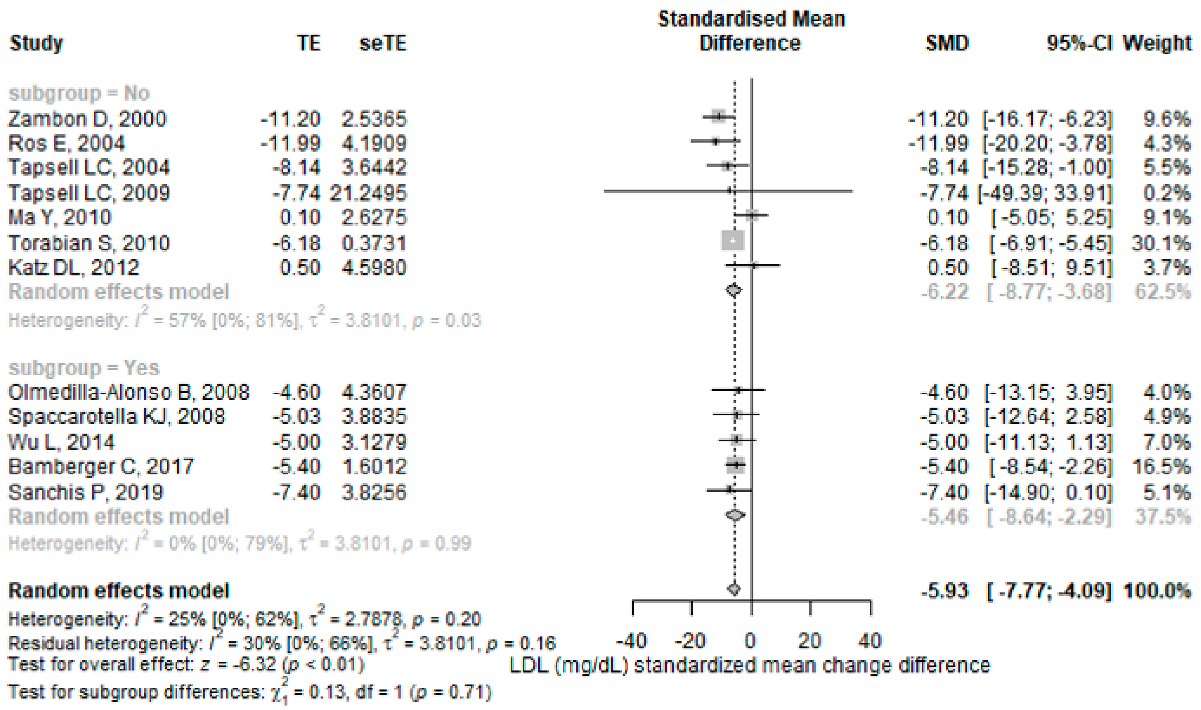

Figure S19. Forest plot for **LDL-C** (mg/dL) standardized mean change difference, comparing with subgroup analyses for 40 years of age or mean age ≥ 50 years.

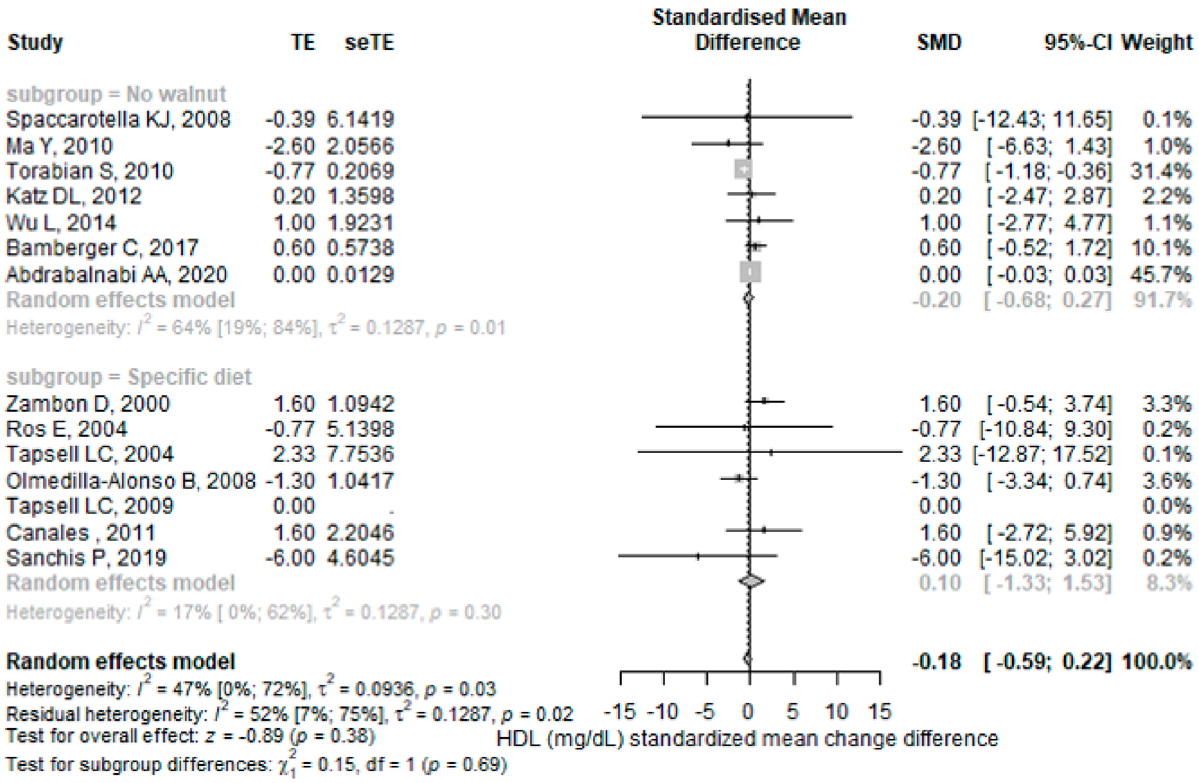

Figure S20. Forest plot for **HDL-C** (mg/dL) standardized mean change difference, with subgroup analyses for Control.

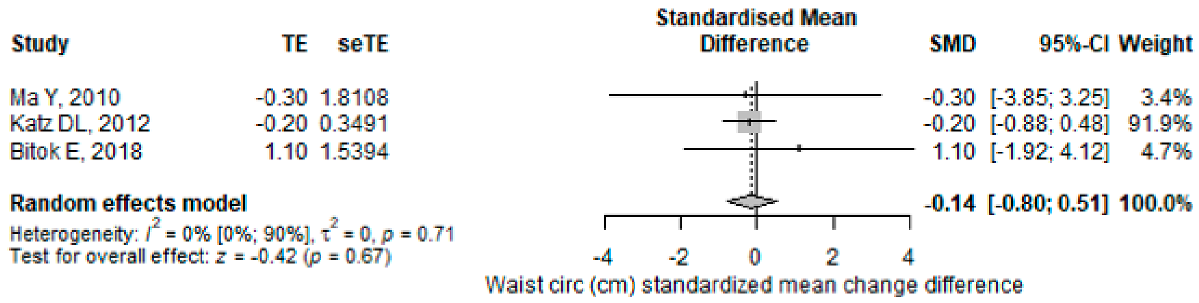

Figure S21. Forest plot for **WC** (cm) standardized mean change difference.

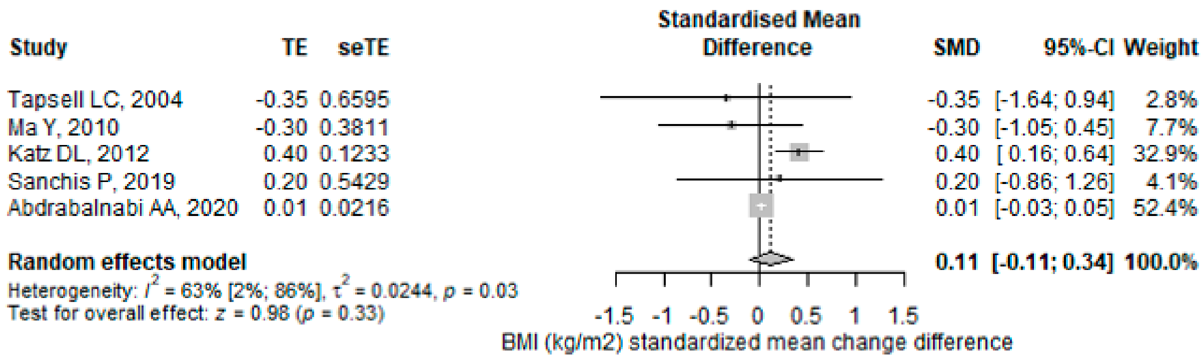

Figure S22. Forest plot for **BMI** (kg/m2) standardized mean change difference.

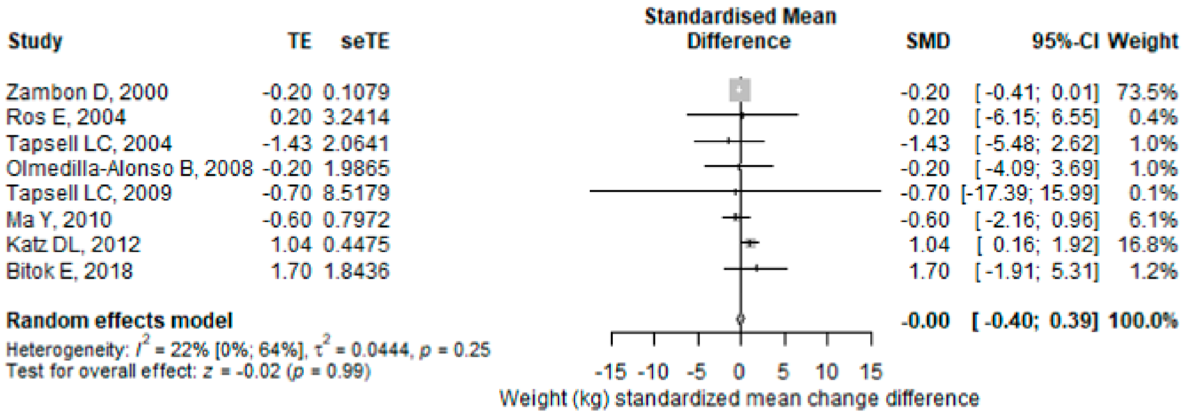

Figure S23. Forest plot for **BW** (kg) standardized mean change difference.

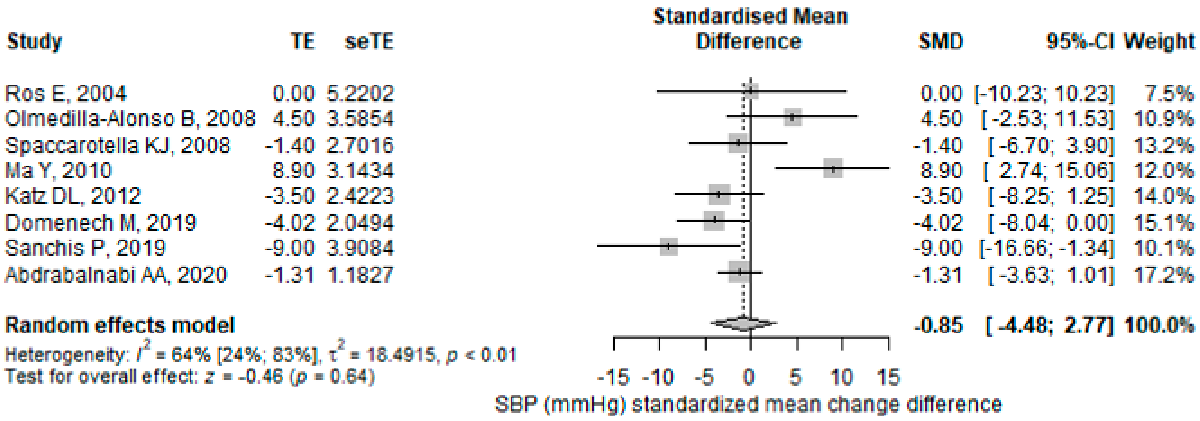

Figure S24. Forest plot for **SBP** (mmHg) standardized mean change difference.

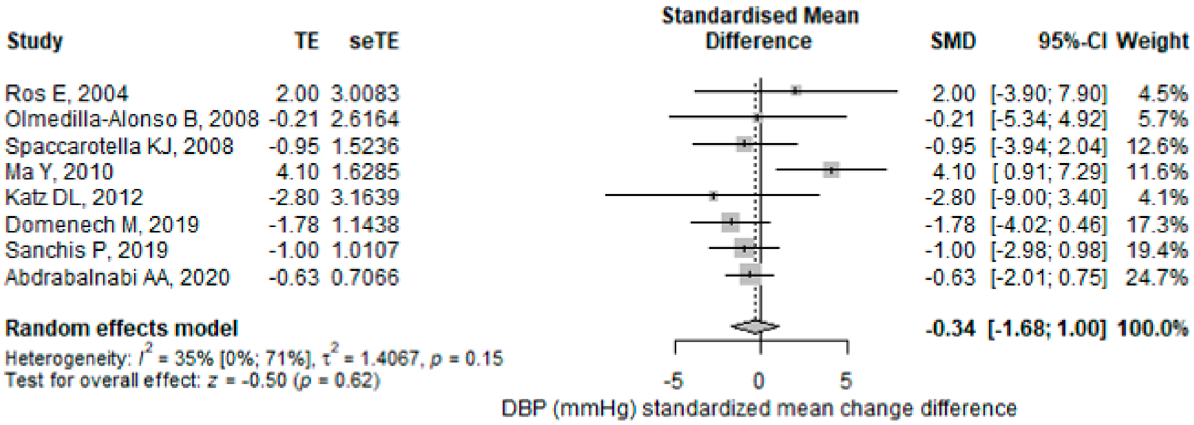

Figure S25. Forest plot for **DBP** (mmHg) standardized mean change difference.

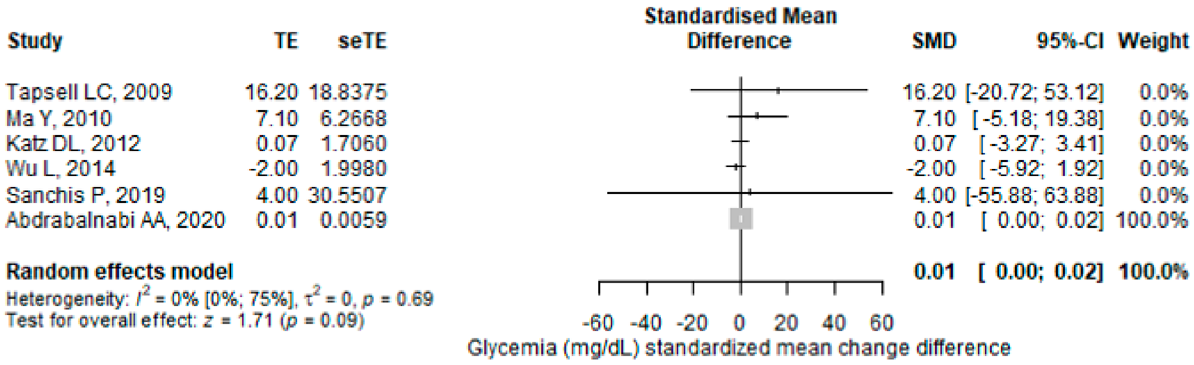

Figure S26. Forest plot for **FBG** (mg/dL) standardized mean change difference.

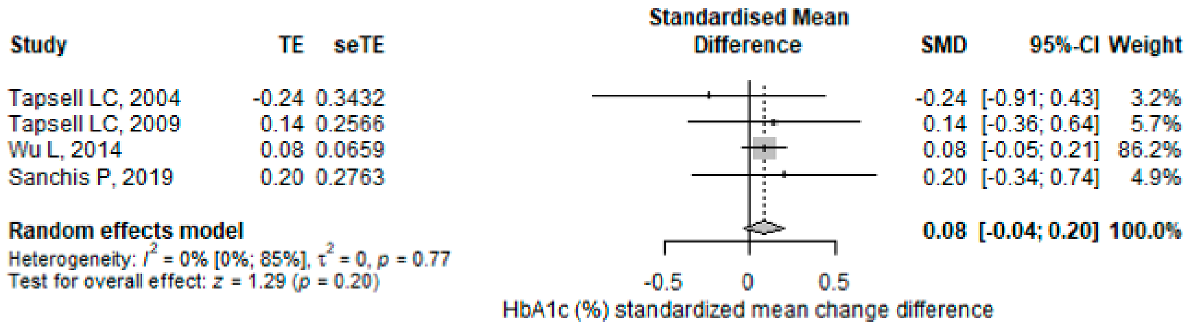

Figure S27. Forest plot for **HbA1c** (%) standardized mean change difference.

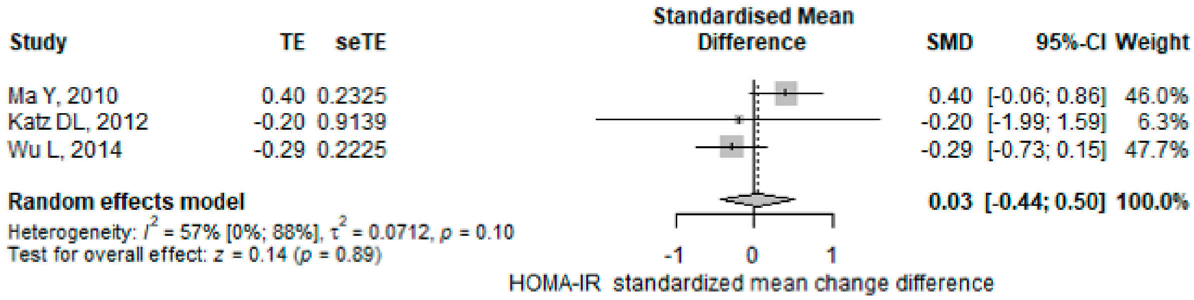

Figure S28. Forest plot for **HOMA-IR** standardized mean change difference.

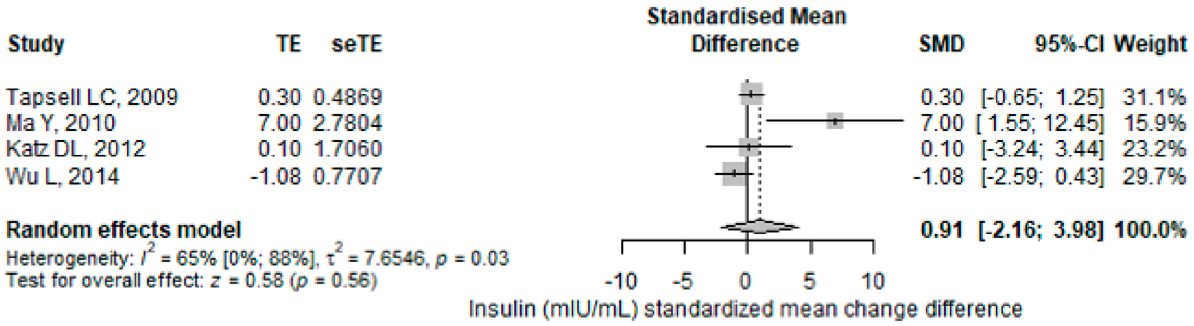

Figure S29. Forest plot for **Insulin** (mIU/mL) standardized mean change difference.

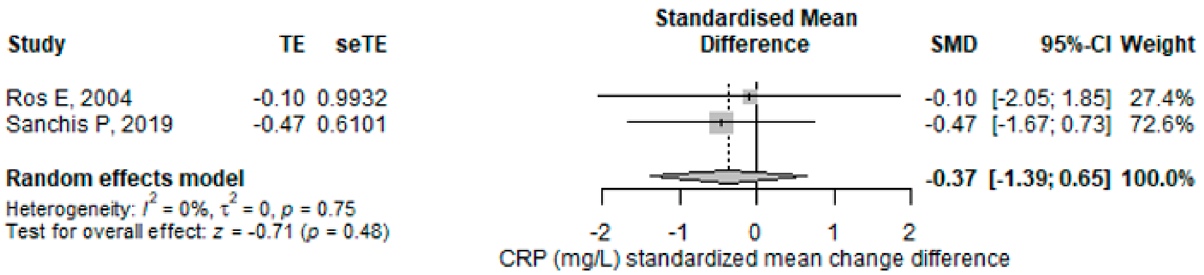

Figure S30. Forest plot for **CRP** (mg/L) standardized mean change difference.

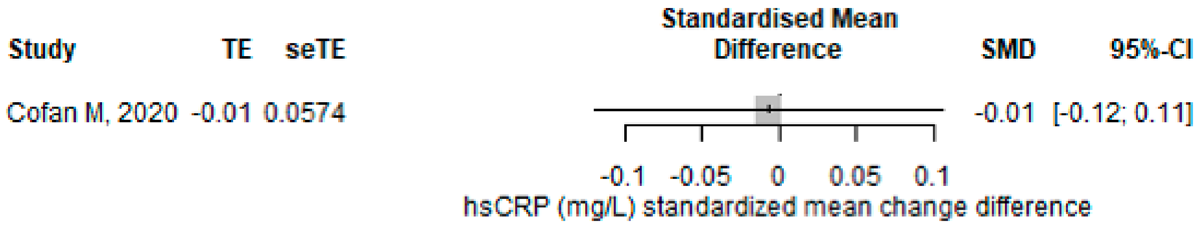

Figure S31. Forest plot for **hs-CRP** (mg/L) standardized mean change difference.

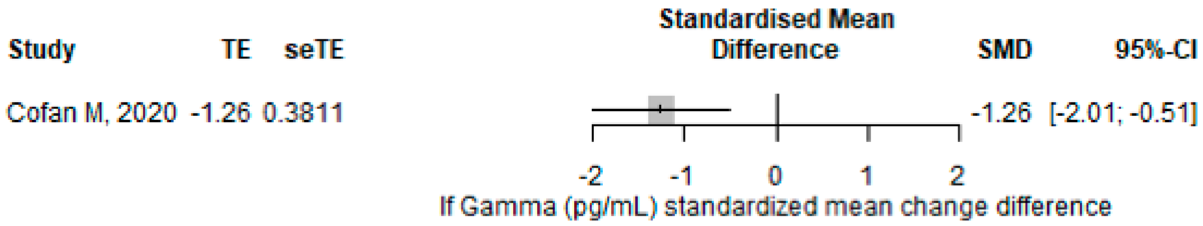

Figure S32. Forest plot for IFN-γ (pg/mL) standardized mean change difference.

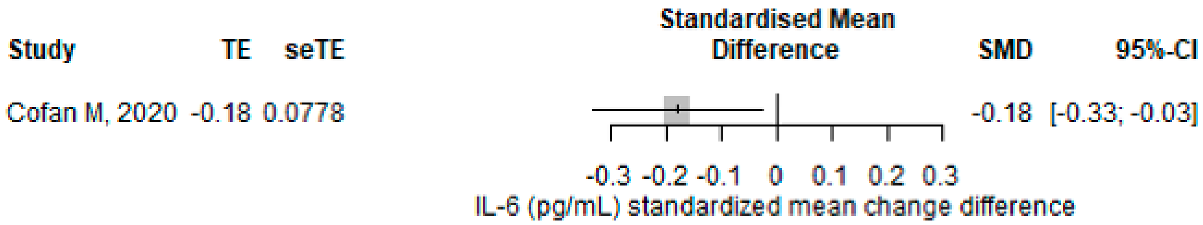

Figure S33. Forest plot for **IL-6** (pg/mL) standardized mean change difference.

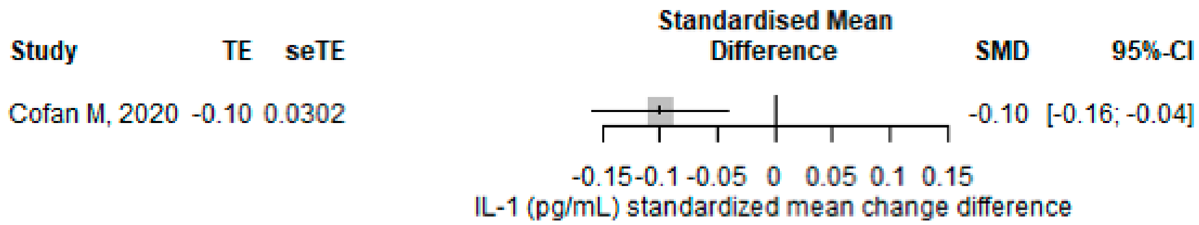

Figure S34. Forest plot for **IL-1β** (pg/mL) standardized mean change.

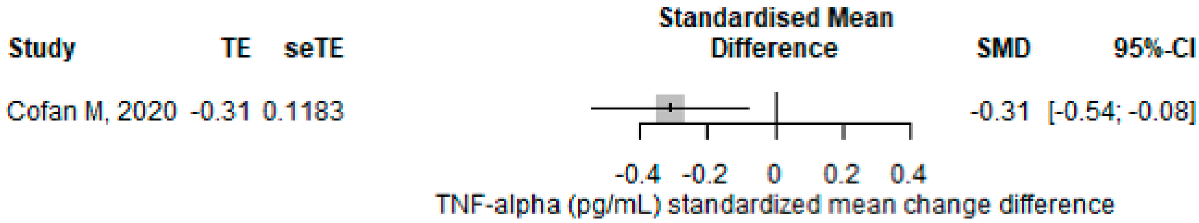

Figure S35. Forest plot for **TNF-** α (pg/mL) standardized mean change difference.

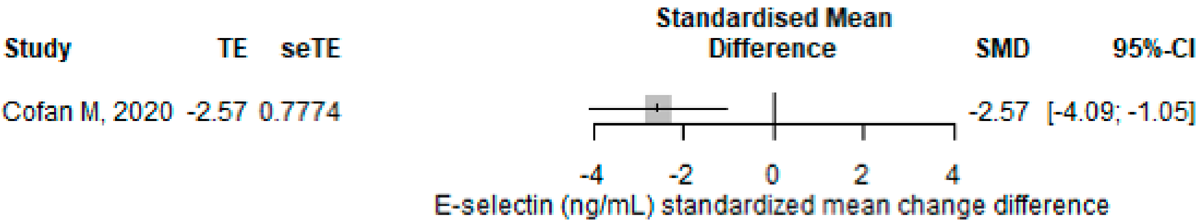

Figure S36. Forest plot for **E-selectin** (ng/mL) standardized mean change difference.

Risk of bias assessment

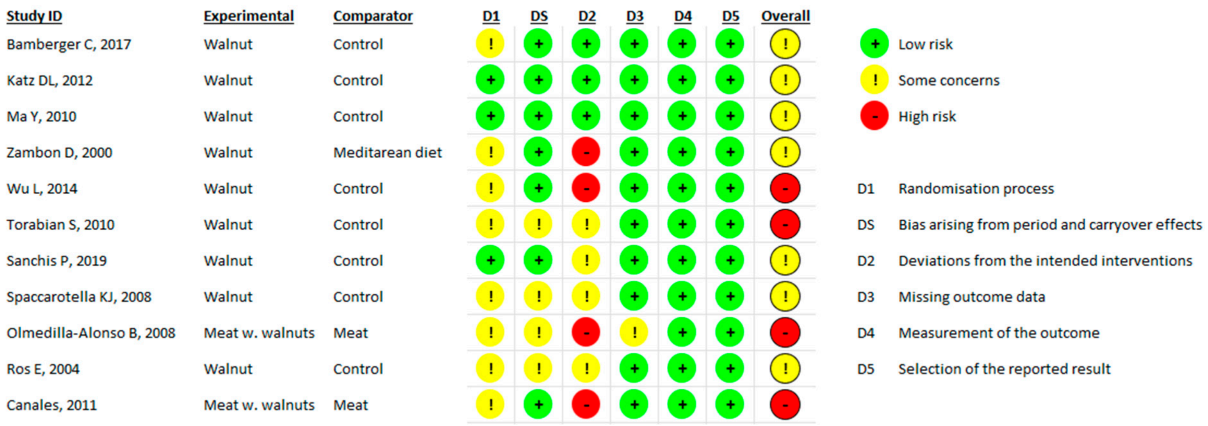

Figure S37. Risk of bias assessment for crossover randomized controlled trials.

Study ID	Experimental	Comparator	D1	D2	D3	D4	D5	Overall
Tapsell, 2004	Walnut	Control	!	+	+	+	+	!
Tapsell, 2009	Walnut	Control	!	!	+	+	+	!
WAHA *	Walnut	Control	!	!	+	+	+	+

+ Low risk

! Some concerns

- High risk

D1 Randomisation process

D2 Deviations from the intended interventions

D3 Missing outcome data

D4 Measurement of the outcome

D5 Selection of the reported result

Figure S38. Risk of bias assessment for parallel randomized controlled trials. [*WAHA * - Walnuts and Healthy Aging*. Several papers analyzed data from the same study, and here it is assessed only once.]
